# A mixed‐studies systematic review of the experiences of body image, disordered eating, and eating disorders during the COVID‐19 pandemic

**DOI:** 10.1002/eat.23706

**Published:** 2022-03-23

**Authors:** Jekaterina Schneider, Georgina Pegram, Benjamin Gibson, Deborah Talamonti, Aline Tinoco, Nadia Craddock, Emily Matheson, Mark Forshaw

**Affiliations:** ^1^ Centre for Appearance Research, Department of Health and Social Sciences University of the West of England Bristol UK; ^2^ School of Psychology, Faculty of Health Liverpool John Moores University Liverpool UK; ^3^ Research Centre and Centre EPIC Montreal Heart Institute Montreal Quebec Canada

**Keywords:** body image, coronavirus, COVID‐19, disordered eating, eating and feeding disorders, health inequality, isolation, lockdown, narrative synthesis, pandemic

## Abstract

**Objectives:**

This systematic review assessed the influence of the COVID‐19 pandemic and associated restrictions on body image, disordered eating (DE), and eating disorder outcomes.

**Methods:**

After registration on PROSPERO, a search was conducted for papers published between December 1, 2019 and August 1, 2021, using the databases PsycINFO, PsycARTICLES, CINAHL Plus, AMED, MEDLINE, ERIC, EMBASE, Wiley, and ProQuest (dissertations and theses).

**Results:**

Data from 75 qualitative, quantitative, and mixed‐methods studies were synthesized using a convergent integrated approach and presented narratively within four themes: (1) disruptions due to the COVID‐19 pandemic; (2) variability in the improvement or exacerbation of symptoms; (3) factors associated with body image and DE outcomes; (4) unique challenges for marginalized and underrepresented groups. Disruptions due to the pandemic included social and functional restrictions. Although most studies reported a worsening of concerns, some participants also reported symptom improvement or no change as a result of the pandemic. Factors associated with worse outcomes included psychological, individual, social, and eating disorder‐related variables. Individuals identifying as LGBTQ+ reported unique concerns during COVID‐19.

**Discussion:**

There is large variability in individuals' responses to COVID‐19 and limited research exploring the effect of the pandemic on body image, DE, and eating disorder outcomes using longitudinal and experimental study designs. In addition, further research is required to investigate the effect of the COVID‐19 pandemic on body image and eating concerns among minoritized, racialized, underrepresented, or otherwise marginalized participants. Based on the findings of this review, we make recommendations for individuals, researchers, clinicians, and public health messaging.

**Public Significance:**

This review of 75 studies highlights the widespread negative impacts that the COVID‐19 pandemic and associated restrictions have had on body image and disordered eating outcomes. It also identifies considerable variations in both the improvement and exacerbation of said outcomes that individuals, researchers, clinicians, and other public health professionals should be mindful of if we are to ensure that vulnerable people get the tailored support they require.

## Abstract (es)

**Objetivos:**

Esta revisión sistemática evaluó la influencia de la pandemia de COVID‐19 y las restricciones asociadas en los resultados en imagen corporal, la alimentación disfuncional y los trastornos alimentarios.

**Método:**

Después del registro en PROSPERO, se realizó una búsqueda de artículos publicados entre el 1 de diciembre de 2019 y el 1 de agosto de 2021, utilizando las bases de datos PsycINFO, PsycARTICLES, CINAHL Plus, AMED, MEDLINE, ERIC, EMBASE, Wiley y ProQuest (disertaciones y tesis).

**Resultados:**

Los datos de 75 estudios cualitativos, cuantitativos y de métodos mixtos se sintetizaron utilizando un enfoque integrado convergente y se presentaron narrativamente dentro de cuatro temas: (1) interrupciones debidas a la pandemia de COVID‐19; (2) variabilidad en la mejoría o exacerbación de los síntomas; (3) factores asociados con resultados de la imagen corporal y alimentarios disfuncional; (4) desafíos únicos para los grupos marginados y subrepresentados. Las interrupciones debidas a la pandemia incluyeron restricciones sociales y funcionales. Aunque la mayoría de los estudios informaron un empeoramiento de las preocupaciones, algunos participantes también informaron una mejoría de los síntomas o ningún cambio como resultado de la pandemia. Los factores asociados con peores resultados incluyeron variables psicológicas, individuales, sociales y relacionadas con el trastorno alimentario. Las personas que se identificaron como LGBTQ + informaron preocupaciones únicas durante COVID‐19.

**Discusión:**

Existe una gran variabilidad en las respuestas de los individuos a COVID‐19 y una investigación limitada que explora el efecto de la pandemia en los resultados de la imagen corporal, la alimentación disfuncional y los trastornos de la conducta alimentaria utilizando diseños de estudios longitudinales y experimentales. Además, se requiere más investigación para investigar el efecto de la pandemia de COVID‐19 en la imagen corporal y las preocupaciones alimentarias entre los participantes minoritarios, racializados, subrepresentados o marginados. Basados en los hallazgos de esta revisión, se hacen recomendaciones para individuos, investigadores, médicos y mensajes de salud pública.

## 1 INTRODUCTION

The novel coronavirus disease (COVID‐19) is an infectious respiratory illness that has claimed millions of lives globally (World Health Organization, 2021) and has had significant psychological ramifications. Individuals with disordered eating (DE) and eating disorders (EDs) may be particularly vulnerable due to the impact of distancing measures on social support and access to mental health services (Christensen, Hagan, et al., 2021; Touyz et al., 2020), as well as their problematic relationships with food in a time of food insecurity (Islam et al., 2021). Emerging evidence suggests that public health messaging associated with COVID‐19 and increased reliance on videoconferencing technologies have had a negative impact on body image (BI; Pearl & Schulte, 2021; Pikoos et al., 2021), which is closely associated with DE and ED symptoms (Smolak & Levine, 2015). A systematic investigation is therefore warranted to explore the ways in which the ongoing pandemic might impact BI, DE, and ED outcomes.

### 1.1 Lockdown and social distancing measures

Previous reviews have documented the negative impact of social restrictions (e.g., “lockdowns” and related physical distancing measures) associated with the COVID‐19 pandemic on psychological well‐being (Brooks et al., 2020; Rajkumar, 2020; Schneider et al., 2021). Early commentary has indicated that social restrictions may be particularly challenging for individuals living with, and vulnerable to, EDs due to changes in daily routine and increased psychological distress (Rodgers et al., 2020). Furthermore, there have been notable disruptions to treatment and access to professional support (Weissman et al., 2020), though some studies have highlighted a potential benefit of increased online service provision for those not already connected with care (Simpson et al., 2021). In addition, studies have found that changes to meal patterns, food planning and buying, and physical activity have negatively impacted cognitions and behaviors across the spectrum of ED diagnoses (Hansen & Menkes, 2021; Hayes & Smith, 2021). Individuals with higher levels of psychological distress related to COVID‐19 restrictions are more likely to report increased BI concerns and DE (Flaudias et al., 2020; Swami, Horne, et al., 2021), as well as worsened ED symptomatology (Baenas et al., 2020; Chan & Chiu, 2021). This may be exacerbated by increased social media use due to limits on in‐person interactions (Pikoos et al., 2021) and increased exposure to content related to eating and appearance (Holland & Tiggemann, 2016).

### 1.2 Harmful messaging around quarantine weight gain

Research has also highlighted harmful messaging around weight gain during the COVID‐19 pandemic. Terms such as “covibesity” (Khan & Smith, 2020), “COVID‐15” (Pearl, 2020), and “Quarantine‐15” (Pearl & Schulte, 2021) are considered new risk factors for ED cognitions and behaviors, as well as increased BI concerns. Although considerable research has been conducted on the prevalence of weight gain during COVID‐19, less research has considered the exposure effects associated with this messaging (Pearl & Schulte, 2021). Previous literature shows a negative impact of weight stigmatizing public health messages on multiple physical and mental health outcomes, including reduced physical activity, increased binge eating, greater psychological distress, and an increase in body dissatisfaction (Bristow et al., 2020; Emmer et al., 2020; Mensinger et al., 2021). As such, media coverage of COVID‐19 related to weight gain is likely to exacerbate weight stigma and psychological distress in individuals with EDs, as well as DE behaviors and BI concerns in the general population (Lessard & Puhl, 2021).

### 1.3 Food insecurity and food shortages

Food insecurity (i.e., concern about, or actual changes in, food availability) has been associated with binge eating and binge eating disorder (BED), bulimia nervosa (BN), as well as general ED pathology and symptomatology (Becker et al., 2017; Hazzard et al., 2020; Lydecker & Grilo, 2019; Rasmusson et al., 2019; Zickgraf et al., 2022). Food insecurity may be exacerbated during COVID‐19 due to increased financial stress and economic limitations, as well as the “panic buying” behaviors and shortage of staple foods that characterized the initial phases of the pandemic (Khosravi, 2020; Weissman et al., 2020). These effects may be further strengthened by the pervasive media coverage about threats of food shortages (Rasmusson et al., 2019). Early research has shown increased food insecurity across multiple countries as a result of the pandemic (Mishra & Rampal, 2020; Niles et al., 2020; Zidouemba et al., 2020), but fewer studies have explored the influence of food insecurity on eating outcomes (Christensen, Forbush, et al., 2021; Coulthard et al., 2021).

### 1.4 Eating and BI concerns in marginalized groups

To date, most BI and ED research has been conducted with predominantly White, cisgender, and heterosexual participants, with the vast majority of studies taking place in Western, educated, industrialized, rich, and democratic (WEIRD) countries (Mikhail & Klump, 2021). ED research is often conducted with female samples, thus underrepresenting men and nonbinary or genderqueer participants (Burke et al., 2020). This is despite evidence showing that participants who identify as Black, Indigenous, or other People of Color (BIPOC) and participants from non‐WEIRD countries experience equivalent, if not higher, rates of EDs (Acle et al., 2021; Alfalahi et al., 2021). In addition, recent studies have found higher prevalence of BI and DE concerns in lesbian, gay, bisexual, transgender, and questioning or queer (LGBTQ+) participants (Nowaskie et al., 2021). Such effects are compounded among individuals who experience multiple intersecting inequalities (e.g., Black, LGBTQ+ women; Crenshaw, 2017), and are thus likely to show the highest prevalence of BI concerns and DE (Beccia et al., 2021). A recent review on the impact of inequality factors on mental health outcomes during COVID‐19 found that certain individual characteristics, such as female gender, existing psychological health conditions, and being subjected to stigma because of one's identity as a member of an ethnic or sexual minority group predicted worse mental health outcomes (Gibson et al., 2021). However, to date, no reviews have looked specifically at BI and eating outcomes during COVID‐19 among marginalized and underrepresented populations.

### 1.5 The current review

The current mixed‐studies systematic review aimed to assess the influence of COVID‐19 on BI, DE behaviors, and ED outcomes. Several reviews have investigated the effect of COVID‐19 on EDs (Miniati et al., 2021; Monteleone, Cascino, Barone, et al., 2021; Sideli et al., 2021), reporting worsening of symptoms, increased levels of anxiety, and difficulties in treatment compliance during lockdown. However, little is still known about the adverse effects of the pandemic on ED outcomes and no systematic review has hitherto considered the influence of COVID‐19 on BI and DE behaviors in the general population. Furthermore, no previous reviews have adopted a mixed‐studies approach to assess the influence of COVID‐19 on BI and eating concerns. Mixed‐studies reviews maximize the findings of traditional systematic reviews (i.e., reviews summarizing qualitative *or* quantitative studies), and thus the ability of those findings to inform policy and practice (Harden, 2010; Stern et al., 2020). As such, the current mixed‐studies review will provide a more comprehensive depiction of the influence of COVID‐19 on BI and eating outcomes, and enhance the utility and impact of findings (Harden & Thomas, 2010; Noyes et al., 2019).

Specifically, we aimed to: (1) identify studies that assess the influence of the COVID‐19 pandemic and related variables (e.g., experience of quarantine, social distancing measures, “stay at home” orders, lockdown) on BI, DE, and EDs; (2) evaluate the influence of the COVID‐19 pandemic on ED‐specific and general psychopathology in individuals with EDs; (3) explore possible differences in the experiences of, and responses to, the pandemic among participants from marginalized and underrepresented populations; and (4) provide evidence‐based recommendations for individuals, researchers, clinicians, and public health messaging.

## 2 METHODS

This review was conducted in collaboration with researchers who have lived experience of EDs (J.S., G.P., and N.C.), clinical experience in nutrition and EDs (A.T. and E.M.), and research expertise in health psychology (B.G. and M.F.), BI (J.S., G.P., A.T., N.C., and E.M.), EDs (J.S., G.P., N.C., D.T., and E.M.), public health (B.G. and M.F.), and conducting systematic reviews (J.S., B.G., D.T., E.M., and M.F.). The review follows the updated Preferred Reporting Items for Systematic Reviews and Meta‐Analyses statement (PRISMA; Page et al., 2021) and was preregistered on PROSPERO (ref no. CRD42021247921) prior to commencement (May 10, 2021).

### 2.1 Data sources and search strategy

Searches were conducted for papers published between December 1, 2019 and August 1, 2021, using the databases PsycINFO, PsycARTICLES, CINAHL Plus, AMED, MEDLINE, ERIC (all accessed via EBSCO), EMBASE, Wiley, and ProQuest (dissertations and theses). We did not limit searches by language or country of publication. Boolean combinations of the following search terms and their abbreviations were used: anorexia; appearance anxiety; appearance comparison; appearance concern; atypical anorexia; atypical bulimia; atypical eating disorder; binge; binge eating; body anxiety; body checking; body dissatisfaction; body dysmorphia; body dysmorphic disorder; body image; body image concern; bulimia; compulsive exercise; dietary restriction; disordered eating; eating disorder; eating pathology; eating disorders not otherwise specified; excessive exercise; feeding disorder; food intake disorder; food restriction; laxative; obsessive exercise; orthorexia; other specified feeding and eating disorders; over exercising; pica; purging; restrictive diet; rumination disorder; shape concern; vomit; weight concern; coronavirus; COVID; COVID‐19; lockdown; pandemic; quarantine; SARS‐CoV‐2; social distancing. The full search strategy can be found on the project's Open Science Framework page (https://osf.io/pz48w/).

Reference sections of the included articles were scanned to identify additional studies that met inclusion criteria. We also searched for “gray literature” and unpublished studies uploaded to PsyArXiv or registered on ClinicalTrials.gov, as well as conference abstracts (*Appearance Matters 9 Online Conference*, July 13–15, 2021; *International Conference on Eating Disorders*, June 10–12, 2021) to ensure we captured all relevant research, given the timeliness of COVID‐19. In addition, Emeritus Professor Michael Levine forwarded our request for published and unpublished research to the Levine Prevention/Sociocultural Factors TinyLetter email group, consisting of approximately 1060 researchers across 49 countries who are actively involved in BI and ED research.

### 2.2 Study eligibility criteria

We included papers that examined the influence of the COVID‐19 pandemic or variables directly related to the pandemic (e.g., experience of quarantine, social distancing measures, “stay at home” orders, lockdown, COVID‐19‐related anxiety or stress) on BI, DE, and ED outcomes. BI outcomes included, for example, experiences of weight stigma, appearance concerns, and body anxiety. DE and ED outcomes included onset of, exacerbation of, or change in specific symptoms (e.g., binge eating, purging, compulsive exercise, food restriction), mental health and well‐being in individuals with diagnosed or undiagnosed EDs (e.g., anxiety, stress, depression, psychological distress, negative affect), and treatment outcomes (e.g., adherence to treatment, treatment efficacy). This was a mixed‐studies review; as such, we included controlled trials, cohort studies, cross‐sectional studies, case reports, and qualitative studies that examined the influence of the COVID‐19 pandemic on target outcomes. We excluded: (1) review papers, commentaries, opinion pieces, and editorials; (2) studies that did not assess the direct relationship between the COVID‐19 pandemic and target outcomes (e.g., studies that were conducted during the pandemic, but did not examine the influence of COVID‐19 or related predictors on target outcomes, or studies that did not compare current levels of BI concerns or ED symptoms with prepandemic levels); and (3) studies related to eating habits, dieting, or exercise unrelated to BI or ED symptomatology (e.g., adherence to a Mediterranean diet, changes in physical activity in the general population).

### 2.3 Study selection

Two authors (J.S. and G.P.) screened titles and abstracts of retrieved papers against the inclusion and exclusion criteria outlined above. Duplicates and irrelevant papers were removed. J.S. and A.T., and G.P. and B.G. independently screened 50% of the full texts that were identified as potentially eligible. The authors held regular meetings to discuss uncertainties and clarify eligibility criteria. Any discrepancies in selecting the final papers for inclusion were resolved through discussion and consultation with the full review team.

### 2.4 Data extraction

Data extraction was completed by four researchers who cross‐checked each other's data extraction (J.S., A.T., G.P., and B.G.). In line with Harden (2010), we used two separate protocols for quantitative and qualitative data. Data extracted for quantitative and qualitative studies are presented in Tables 1 and 2, respectively. For studies that described statistically significant outcomes, a *p* < .05 was considered significant.

**TABLE 1:** Characteristics of quantitative studies

Author(s) (year)	Country	Participants						Methods		Key findings	Study quality
		*N* (% female)^a^	Age *M* (*SD*)	Race and ethnicity	Sexual orientation	Socioeconomic status	Diagnosis	Design	Measures		
Akgül et al. (2021)	Turkey	38 (95%)	15.1 (1.6)	Not reported	Not reported	Not reported	AN‐R *n* = 26; AN‐BP *n* = 5; AAN *n* = 3; BN *n* = 3; OSFED *n* = 1	Cross‐sectional	ED examination; depression; anxiety; obsessive–compulsive symptoms	42% reported improved ED symptomatology, 37% reported no change, 21% reported worse ED symptomatology from pre‐ to during lockdown; 24% reported that lockdown affected access to ED‐related healthcare; depression score had the highest predictive value for ED behavior (*r* ^2^ = .537)	3
Baceviciene and Jankauskiene (2021)	Lithuania	230 (79%)	23.9 (5.4)	Not reported	Not reported	Not reported	n/a	Longitudinal	Attitudes toward appearance; BI; ED examination; self‐esteem	No change in DE or self‐esteem from pre‐ to during lockdown; body appearance evaluation (women only), media pressures (women only), and internalization of thin/low body fat appearance standards (men: *d* = 1.46; women: *d* = 1.18) increased from pre‐ to during lockdown	3
Baenas et al. (2020)	Spain	74 (96%)	32.1 (12.8)	Not reported	Not reported	Not reported	AN *n* = 19; BN *n* = 12; BED *n* = 10; OSFED *n* = 33	Cross‐sectional	ED inventory; food addiction; symptom checklist; temperament and character; telephone survey	26% reported worsened symptoms during confinement (highest in AN and OSFED), 74% reported improvements or no change; patients with worsened symptoms reported lower self‐directedness (*d* = 0.51) and higher prevalence of future concerns (*d* = 0.51), nonadaptive reactions (*d* = 0.79), symptoms of anxiety (*d* = 0.89) and depression (*d* = 0.96), adverse situations (*d* = 0.62), and familiar conflict (*d* = 0.68)	2
Bellapigna et al. (2021)	United States	239 (gender: 79% women; 1.3% nonbinary/nonconforming)	24.7 (11.1)	6.7% Black/African American; 10% Hispanic; 5.9% Asian; 67.8% White; 9.2% multiracial/biracial; 0.4% “other”	Not reported	Education: 25.6% high school degree or equivalent; 46.2% some college; 10.1% 2‐year degree; 11.8% bachelor's degree; 3.4% master's degree; 2.1% doctorate; 2.8% other	6.3% diagnosed with ED	Cross‐sectional	Need for structure; loneliness; social networking; body appreciation; eating attitudes; social phobia; patient health	64% reported more disturbances in BI during COVID; loneliness (*β* = −.138), negative BI (*β* = .253), and social media exposure (*β* = −.161) predicted DE; loneliness (*β* = −.485), negative BI (*β* = .123), and social media exposure (*β* = −.154) predicted depressive symptoms	3
Branley‐Bell and Talbot (2020)^b^	United Kingdom	129 (94%)	29.3 (9.0)	Not reported	Not reported	Not reported	62% current ED/relapse; in recovery 6.2% <3 m, 6.2% 3–12 m, 25.6% >12 m	Cross‐sectional	Mental well‐being; perceived stress; social support; sense of control; rumination	87% reported worsened symptoms as a result of COVID, 30% reported symptoms were much worse, 2% reported slight improvement, 9% reported no change; changes in living situation, social isolation, usual support network(s), physical activity, and time spent online impacted ED symptoms	3
Branley‐Bell and Talbot (2021)^b^	United Kingdom	58 (98%)	30.9 (11.1)	Not reported	Not reported	Not reported	63.8% current ED/relapse; 36.2% in recovery; AN *n* = 28; BN *n =* 7; OSFED *n =* 3; BED *n* = 2; symptoms of multiple EDs *n* = 12; undisclosed ED *n* = 7	Longitudinal	Mental well‐being; perceived stress; social support; sense of control; rumination	15.5% reported relapsing, 19% reported recovering, and 65.5% reported no change in ED status from during to postlockdown; higher perceived control associated with recovery	2
Breiner et al. (2021)	United States	159 (91%)	27.6 (11.7)	90.6% White; 5% Hispanic/Latino; 6.3% Asian; 0.6% American Indian or Alaska Native; 0.6% Native Hawaiian or Pacific Islander; 1.3% Native American	74.8% heterosexual; 2.5% homosexual; 14.5% bisexual	Education: 5% high school graduate; 8.8% less than 2 years of college; 0.6% technical or vocational program; 6.9% associate degree; 62.3% college graduate; 14.5% master's degree; 1.9% doctorate	AN *n* = 22; BN *n* = 8; BED *n =* 4; “other” *n* = 3	Cross‐sectional (retrospective)	ED examination; exercise behaviors; exercise motives; ED diagnosis	No significant changes in exercise or eating pathology from pre‐ to during COVID; eating pathology increased in participants with a prior ED diagnosis (*d* = 0.26), but decreased in participants without a prior ED diagnosis (*d* = −0.14); there were decreases in episodes of eating more than usual (*d* = −0.23) and loss of control over eating (*d* = −0.23), but no changes in objective binge eating (*d* = −0.04), self‐induced vomiting (*d* = −0.05), or laxative use (*d* = −0.14); participants reported increased endorsement of pressure to get in shape from pre‐ to during the pandemic	3
Buckley et al. (2021)^b^	Multiple	204 (86%)	27.0 (8.1)	Not reported	Not reported	Not reported	10.7% current ED; 32.8% previous diagnosis (AN *n* = 29; BN *n* = 11; ON *n* = 9; BED *n* = 7; “other” *n* = 11)	Cross‐sectional	Eating attitudes; BI and food relationship	34.8% reported worse BI, 50.5% reported no change, and 14.6% reported better BI from pre‐ to during COVID; 32.8% reported worse relationship with food, 53.0% reported no change, and 14.1% reported better relationship with food from pre‐ to during COVID	3
Castellini et al. (2020)	Italy	Patients/healthy controls: 74/97 (sex assigned at birth: 100/100% female)	Patients/healthy controls: 31.7/30.5 (12.8/10.9)	100% White	Not reported	Not reported	AN *n* = 37; BN *n* = 37	Longitudinal	Brief symptom inventory; ED examination; psychological distress	Patients reported increased compensatory exercise during lockdown (AN: *d* = 0.32; BN: *d* = 0.30); patients with BN/previously remitted patients reported increased binge eating after lockdown (*d* = 0.32); household arguments (*d* = 0.62) and fear for safety of loved ones (*d* = 0.67) predicted increased symptoms; patients with BN reported more severe COVID‐related posttraumatic symptoms than patients with AN and healthy controls, predicted by childhood trauma (*β* = .34) and insecure attachment (*β* = .57)	1
Cecchetto et al. (2021)	Italy	365 (73%)	35.1 (13.6)	Not reported	Not reported	Not reported	n/a	Cross‐sectional	Binge eating screener; alexithymia; anxiety; eating behaviors; patient health; perceived stress	Binge eating and emotional eating decreased from during to postlockdown; emotional eating was predicted by higher depression, anxiety, lower quality of personal relationships, and lower quality of life; increase in binge eating was predicted by higher stress	2
Chan and Chiu (2021)	Hong Kong	316 (71%)	25.1 (5.0)	Not reported	Not reported	88.3% university educated	n/a	Cross‐sectional	ED screening; patient health; anxiety; psychological well‐being; eating behaviors and emotions	Among individuals with elevated depression, those who attributed depression to COVID reported higher levels of symptoms; no effect on anxiety	3
Christensen, Forbush, et al. (2021)	United States	579 (gender identity: 76% women; 2% “other”)	21.8 (5.3)	9% Hispanic; 91% non‐Hispanic; 84.1% White; 3.5% Black/African American; 1% American Indian/Alaskan Native; 5.5% Asian/Pacific Islander; 5.2% multiracial; 0.7% not disclosed	Not reported	Years of posthigh school: 20.3% <1 year; 18.7% 1 year; 19.4% 2 years; 22.0% 3 years; 9.9% 4 years; 2.4% 5 years; 5.0% 6+ years 5%; 2.3% continuing education; food insecurity: 52.8% none; 6.6% household food insecurity; 40.6% individual food insecurity	AN *n* = 4; BN *n* = 75; BED *n* = 14; OSFED *n =* 135; no ED diagnosis *n* = 344	Cross‐sectional	Clinical impairment; ED diagnosis; food insecurity	Students with food insecurity showed higher prevalence of ED diagnoses and reported greater frequency of objective binge eating, compensatory fasting, and ED‐related impairment compared with individuals without food insecurity; there were no differences in food insecurity before or during the beginning of the COVID pandemic; participants who identified as Black were significantly more likely to report individual food insecurity relative to other racialized groups	2
Conceição et al. (2021)	Portugal	COVID/non‐COVID group: 35/66 (94/83%)	COVID/non‐COVID group: 50.8/50.1 (12.4/10.7)	Not reported	Not reported	Education (COVID/non‐COVID group): ≤6 years (42.9/31.8%; 9–12 years (34.3/48.5%); college degree (22.9/19.7%); professional status: student (2.9/1.5%); employed (62.9/57.6%); unemployed (11.4/25.8%); retired: (22.9/15.2%)	n/a	Cohort	ED examination; repetitive eating; depression, anxiety, stress; impulsivity	Participants assessed post‐COVID showed a greater increase in weight concern scores (*ƞ* ^2^_p_ = 0.094) and repetitive eating (*ƞ* ^2^_p_ = 0.076) compared with participants assessed pre‐COVID; no difference between groups in shape concern, food concerns, or restraint eating	1
Coulthard et al. (2021)	United Kingdom	620 (88%)	39.9 (14.0)	88% White‐British/European; 6% Asian/British Asian; 1% Black/Black British; 4% “other”; 1% not disclosed	Not reported	Occupation: 39% professional; 21% intermediate; 15% manual; 25% not working; food insecurity: 65% none; 29% mild; 6% moderate/severe	n/a	Cross‐sectional	Food consumption; ED symptoms; coping strategies; anxiety; food insecurity	Emotional eating decreased from pre‐ to during lockdown; women and those isolating at home were more likely to report higher emotional eating during lockdown; there was no differences in eating behavior based on occupation or ethnicity; higher emotional eating during lockdown associated with higher BMI, higher prelockdown emotional eating, and maladaptive coping strategies	2
Czepczor‐Bernat et al. (2021)	Poland	671 (100%)	32.5 (11.4)	98.8% White; 0.3% mixed race; 0.9% “other”	91.3% heterosexual; 1.6% lesbian; 4.9% bisexual; 0.7% pansexual/queer; 0.9% asexual; 0.6% “other”	Education: cluster 1 32% secondary/technical school; cluster 2 32% secondary/technical school; cluster 3 37% master's degree; cluster 4 45% master's degree	n/a	Cross‐sectional	COVID‐related stress; COVID‐related anxiety; ED inventory; BI	Higher levels of ED symptoms and negative BI were observed in women with excess body weight, high anxiety, and stress related to COVID as compared with women with a healthy body weight and with low levels of anxiety and stress	3
Félix et al. (2021)	Portugal	24 (100%)	50.9 (12.8)	Not reported	Not reported	Education: 29.2% elementary school; 25.0% middle school; 12.5% high school; 33.3% college degree; employment: 66.7% employed; 8.3% unemployed; 25.0% retired	n/a	Cross‐sectional	Depression, anxiety, stress; impulsivity; DERS; ED examination; loss of control over eating; repetitive eating; ED symptoms; impact on emotions, eating, and weight; COVID impact	Living with fewer people, higher difficulties in dealing with emotionally activating situations, and higher fear of gaining weight during lockdown associated with greater fear of gaining weight, greater fear of losing control over eating, and greater DE psychopathology	2
Fernández‐Aranda, Munguía, et al. (2020)	Spain	121 (86%)	33.7 (15.8)	Not reported	Not reported	Not reported	AN *n* = 55; BN *n* = 18; OSFED *n* = 14	Cross‐sectional	COVID isolation eating scale	Patients with AN reported a positive response to treatment during confinement; no significant changes found in patients with BN; patients with OSFED reported an increase in eating symptomatology and psychopathology; patients with AN reported greatest dissatisfaction and accommodation difficulty with remote therapy	3
Fernández‐Aranda, Casas, et al. (2020) Study 1	Spain	32 (91%)	29.2 (range 16–49 years)	Not reported	Not reported	Not reported	AN *n* = 13; BN *n* = 10; BED *n* = 4; OSFED *n =* 5	Cross‐sectional	Telephone survey	Most patients presented worries about increased uncertainties, such as the risk of COVID infection of themselves or their loved ones, the negative impact on their work, and their treatment; 38% reported impairments in ED symptomatology; 56% reported additional anxiety symptoms	3
Flaudias et al. (2020)	France	5738 (75%)	21.2 (4.5)	Not reported	Not reported	University students, 48.8% with scholarship	38.3% at risk for ED symptoms	Cross‐sectional	Anxiety and depression; perceived stress; ED inventory; ED screening; ideal body stereotypes	Greater likelihood of binge eating (BE) and dietary restriction (DR) over past week and/or future intentions to binge eat (FBE) and restrict (FDR) associated with lockdown‐related stress (all odds ratios [OR]; BE = 1.12; DR = 1.17; FBE = 1.33; FDR = 1.12), exposure to COVID‐related media (BE = 1.02; DR = 1.05; FBE = 1.20; FDR = 1), female gender (BE = 1.40; DR = 1.79; FBE = 1.09; FDR = 1.48), greater levels of anxiety (BE = 1.09; DR = 1.11; FBE = 0.95; FDR = 1.04) and depression (BE = 1.14; DR = 0.94; FBE = 1.40; FDR = .95), low impulse regulation (BE = 1.10; DR = 1.04; FBE = 1.23; FDR = 1.12), higher BMI (BE = 1.26; DR = 1.07; FBE = 1.19; FDR = 1.04), body dissatisfaction (BE = 1.08; DR = 1.80; FBE = 0.84; FDR = 2.05), and concurrent probable ED (BE = 2.82; DR = 2.65; FBE = 2.11; FDR = 2.58)	3
Giel et al. (2021)	Germany	42 (81%)	45.5 (12.6)	Not reported	Not reported	Not reported	BED *n =* 17	Longitudinal	ED examination; perceived stress; binge eating frequency; depression; emotion regulation; coherence	Binge eating frequency (*χ* ^2^ = 15.22), general ED pathology (*χ* ^2^ = 35.52), and depressive symptoms (*χ* ^2^ = 5.41) increased from pre‐ to post‐COVID; individuals scoring high on reappraisal and sense of coherence scored lower on general ED pathology	3
Graell et al. (2020)	Spain	Day hospital/outpatient clinic: 27/338 (93/87%)	Day hospital/outpatient clinic: 13.2/14.7 (3.0/2.3)	Not reported	Not reported	Not reported	ARFID *n* = 48; AN *n* = 255; BN *n* = 26; OSFED *n* = 37	Cross‐sectional (retrospective)	Outpatient and face‐to‐face consultations	42% reported reactivation of ED symptoms following COVID confinement (>adolescents); 68% of patients and their families reported onset of confinement and 41% reported social isolation from peers as influencing factors for admission	2
Gullo and Walker (2021)	United States	143 (gender: 53% ciswomen; 1% transmen; 0% transwomen; 1% nonbinary)	77% 18–44 years	79.7% White: 9.8% Black: 4.2% Asian; 3.5% Latinx: 1.4% mixed; 0.7% Native American; 0.7% “other”	Not reported	Household annual income (USD): 2.8% <15,000; 9.1% 15,000–29,999; 11.9% 30,000–49,999; 23.8% 50,000–75,999; 16.8% 75,000–99,999; 17.5% 100,000–150,000; 14.0% >150,000; 4.2% prefer not to answer	n/a	Cross‐sectional (retrospective)	Depression, anxiety, stress; BI; binge eating; body satisfaction	Time spent videoconferencing increased from pre‐ to post‐COVID; appearance dissatisfaction increased (*β* = .75), but appearance orientation decreased (*β* = .75) following lockdown; no change in binge eating from pre‐ to postlockdown; videoconferencing time did not predict BI or binge eating postlockdown	3
Haddad et al. (2020)	Lebanon	407 (51%)	30.6 (10.1)	Not reported	Not reported	Education: 90.9% university level; 9.1% secondary school or lower	n/a	Cross‐sectional	Boredom; ED examination; quarantine/ confinement stressors; fear of COVID; anxiety; physical activity	Dietary restraint (DR), eating concerns (EC), shape concerns (SC) and weight concerns (WC) were associated with female gender (all *β*; EC = 0.52; SC = 0.19; WC = 0.20), higher anxiety (EC = 0.04; SC = 0.23; WC = 0.19), sense of insecurity (EC = 0.41), greater fear of COVID (DR = 0.02; SC = 0.20; WC = 0.12), higher BMI (DR = 0.05; EC = 0.06; SC = 0.39; WC = 0.41), physical activity (DR = 1.04; EC = 0.43; SC = 0.15; WC = 0.19), and a higher number of adults living together in quarantine/confinement (SC = 0.10; WC = 0.15)	2
Haddad et al. (2021)	Lebanon	407 (51%)	30.6 (10.1)	Not reported	Not reported	Household monthly income (USD): 31.0% no income; 20.4% <1000; 29.1% 1000–2000; 19.3% >2000	n/a	Cross‐sectional	Boredom; ED examination; quarantine/ confinement stressors; fear of COVID; anxiety; physical activity; perceived weight change	Longer confinement duration (OR = 1.07), higher anxiety (OR = 1.05), and higher eating concerns (OR = 1.81) associated with higher weight change perception; greater fear of COVID (OR = 0.96) and higher self‐reported weight change (OR = 0.47) associated with lower weight change perception	2
Jordan et al. (2021)	United States	140 (89%)	39.8 (6.9)	88.4% White	Not reported	82.2% middle to upper‐middle class	n/a	Cross‐sectional	Perceived stress; concern about weight gain; ED examination; emotional eating	Disordered eating associated with concern about weight gain before (**β**=.18) and during (**β**=.32) COVID; stress and concern about weight gain during COVID predicted variance in eating pathology among caregivers (*r* ^2^ = 0.48).	2
Keel et al. (2020)	United States	90 (88%)	19.5 (1.3)	22% Latinx; 78% White; 12% Black/African American; 4% Asian; 1% American; Indian/Alaskan Native; 3% “other”	89% heterosexual	Not reported	n/a	Longitudinal	Weight perception; physical activity; eating; concerns about weight and shape; body, eating, exercise comparisons; ED diagnosis; screen time	Participants reported increased body weight (*d* = 0.23), eating (*d* = 0.54), screen time (*d* = 1.08), and concerns about weight/shape (*d* = 0.93) and eating (*d* = 0.79), and decreased physical activity (*d* = −0.63) from pre‐ to post‐COVID; no change in weight or BMI, but participants reported shifts in body weight perception from pre‐ to post‐COVID	3
H. Kim, Rackoff, et al. (2021)	United States	Pre‐/during COVID: 3643/4970 (sex assigned at birth: 73/70% female; 0.05/0.02% intersex; gender identity: 70/68% women; 4/2% trans, nonconforming, or self‐identify)	Not reported	Pre‐/during COVID: 12/10% Hispanic; 88/90% non‐Hispanic; 72/75% White; 9/6% Black/African American; 10/14% Asian; 0.7/0.5% American Indian or Alaskan Native; 0.4/0.2% Native Hawaiian or Pacific Islander; 8/5% multiracial	Pre‐/during COVID: 71/81% heterosexual; 29/19% lesbian, gay, bisexual, queer, questioning, or self‐identify	Not reported	Pre‐/during COVID: AN *n* = 64/88; BN/BED *n* = 320/643	Longitudinal	Anxiety; posttraumatic stress; patient health; presence of EDs; insomnia; alcohol use	Depression (*χ* ^2^ = 21.67), alcohol use disorder (*χ* ^2^ = 67.26), BN/BED (*χ* ^2^ = 20.83), and comorbidity (*χ* ^2^ = 6.83) were greater during than before COVID; posttraumatic stress disorder was lower during than pre‐COVID (*χ* ^2^ = 5.46); no differences in anxiety, insomnia, AN, or suicidality between pre‐ and during COVID; no effect of gender, ethnicity/racialized group, or sexuality on EDs	2
S. Kim, Wang, et al. (2021)	United States	7317 (59%)	50.6 (16.1)	64.7% non‐Hispanic White; 7.9% non‐Hispanic Black; 16.8% Hispanic; 5.1% non‐Hispanic Asian; 0.9% Native American; 4.5% “other”; 0.2% not disclosed	Not reported	Education: 5.4% <high school; 16.7% high school or less; 22.8% some college; 14.3% associate degree; 24.3% bachelor's degree; 16.5% advanced college degree	Diagnosed ED *n =* 157; unsure about ED status *n* = 122	Longitudinal	Patient health; perceived stress; loneliness	Individuals with EDs/unsure EDs reported higher levels of psychological distress (all *B*; EDs = 2.18; unsure EDs = 2.01), stress (EDs = 1.17; unsure EDs = 2.08), and loneliness (unsure EDs = 0.90) compared to those without EDs; those unsure about their EDs reported initial decreases in stress and loneliness, but started increasing again since institution of virus containment procedures; levels of loneliness among those with EDs increased initially then began to decrease; individuals with EDs showed steady decreases in stress; identifying as Black, older age, and higher education associated with lower psychological distress (Black = −0.59; age = −0.03; education = −0.03), stress (age = −0.04; education = −0.13), and loneliness (Black = −0.10; age = −0.01); female gender and identifying as Asian associated with higher psychological distress (female = 0.61; Asian = 0.29), stress (female = 0.56; Asian = 1.07), and loneliness (female = 0.14)	1
Koenig et al. (2021)	Germany	Pre/postlockdown: 324/324 (69/69%)	14.9 (1.9)	Not reported	Not reported	Family affluence (pre‐/postlockdown): low (1.9/1.9%); medium (24.4/ 21.6%); high (73.9/76.5%)	n/a	Longitudinal	Strengths and difficulties; patient health; weight concerns; ED examination; quality of life; suicidality	No differences between pre‐ and postlockdown samples in emotional and behavioral problems, depression, thoughts of suicide/suicide attempts, ED symptoms, or quality of life	1
Larkin (2021)	United States	290 (not reported)	Range 18–25 years	0.3% American Indian/Alaskan Native; 2.8% Asian; 10.3% Black/African American; 14.5% Hispanic/Latino; 1.7% biracial/multiracial; 80.7% White	Not reported	Not reported	n/a	Cross‐sectional (retrospective)	Physical activity, social media use; subjective well‐being; BI	32.7% increase in negative BI perceptions from pre‐ to post‐COVID	3
Leenaerts et al. (2021)	Belgium	15 (100%)	Median (IQR) = 23 years (21.5–25.5)	87% European; 13% Asian	Not reported	Not reported	BN	Longitudinal	Affect; location; social context; binge eating frequency	Patients reported higher negative affect (*β* = .15), lower positive affect (*β* = −.10), and changes in surroundings and social context (at home: *β* = 3.19; with housemates: *β* = 3.91; with friends: *β* = −2.45; with family: *β* = .99; with partner: *β* = −2.39) from pre‐ to postlockdown; changes of negative affect associated with binge eating frequency during lockdown (*β* = .61)	2
Lessard and Puhl (2021)	United States	452 (gender identity: 55% women; 2% transgender; 1% “other”; sex assigned at birth: not reported)	14.9 (2.1)	69.9% White; 8.2% Black/African American; 8.0% Latinx; 6.6% multiethnic; 5.5% Asian/Pacific Islander; 1.8% “other”	Not reported	72% had a parent with a college degree or higher	n/a	Cross‐sectional	Body dissatisfaction; exposure to weight stigma on social media; experienced weight stigma	53% reported increased exposure to weight stigmatizing social media content; 41% reported increased body dissatisfaction from pre‐ to post‐COVID (>girls; higher weight), 49% reported no change, 10% reported a decrease	3
Lin et al. (2021)	United States	Not reported	Range 8–26 years	Not reported	Not reported	Not reported	Patients with any ED diagnosis	Longitudinal	COVID‐related trends in ED care‐seeking	Inpatient admissions, hospital bed‐days, outpatient inquiries increased over time post‐COVID compared to stable volume pre‐COVID; outpatient assessments decreased initially following COVID‐related limitations, then rebounded	2
Machado et al. (2020)	Portugal	43 (95%)	27.6 (8.5)	Not reported	Not reported	Not reported	AN *n* = 20; BN *n* = 14; BED *n* = 2; OSFED *n =* 7	Longitudinal	ED examination; clinical impairment; impulsivity; difficulties in emotion regulation; COVID impact	Of 26 patients in treatment 31% remained unchanged, 27% deteriorated, and 42% improved; of 17 participants not in treatment 53% remained unchanged, 18% deteriorated, and 29% improved from during to postlockdown; higher impact correlated with ED symptoms, impulsivity (*r* = .380), psychopathology (*r* = .451), emotion regulation difficulties (*r* = .393) and clinical impairment (*r* = .569)	3
Martínez‐de‐Quel et al. (2021)	Spain	161 (37%)	35.0 (11.2)	Not reported	Not reported	Not reported	n/a	Longitudinal	Eating attitudes	No change in ED risk from before to during COVID lockdown	2
Meda et al. (2021)	Italy	358 (80%)	21.3 (2.1)	Not reported	Not reported	Not reported	Not reported	Longitudinal	ED inventory; eating habits; obsessive–compulsive symptoms; anxiety; depression	Only students with ED history reported an increase in ED symptomatology from pre‐ to postlockdown (*β* = .1).	3
Monteleone, Cascino, Marciello, et al. (2021)	Italy	312 (gender identity: 96% women; 0.3% nonbinary)	AN: 26.9 (10.3); other EDs: 32.3 (13.5)	Not reported	Not reported	Employment (AN/other EDs): paid job (26/39%); student (56/49%)	AN *n =* 179; BN *n =* 63; BED *n =* 48; OSFED *n* = 22	Cross‐sectional (retrospective)	Factors related to COVID concerns; illness duration; treatment‐related variables; ED psychopathology	General (GP) and specific psychopathology (SP) worsened from pre‐ to postlockdown; perceived low quality of therapeutic relationships (GP: *β* = −.16; SP: *β* = −.22), fear of contagion and increased isolation (GP: *β* = .22; SP: *β* = .22), reduced satisfaction with relationships (SP: *β* = .24), and reduced social support (GP: *β* = .23) associated with worsened psychopathology; no effect of intimate relationships, illness duration, diagnosis, economic change, or type of treatment	2
Monteleone, Marciello, et al. (2021)	Italy	312 (gender identity: 96% women; 0.3% nonbinary)	29.2 (12.1)	Not reported	Not reported	Not reported	AN *n* = 179; BN *n* = 63; BED *n* = 48; OSFED *n* = 22	Cross‐sectional (retrospective)	Anxiety; posttraumatic stress; obsessive–compulsive symptoms; patient health; ED inventory	General and specific psychopathology worsened from pre‐ to postlockdown; symptoms persisted postlockdown, apart from suicide ideation; individuals with AN reported higher anxiety, obsessive–compulsive symptoms, suicide ideation, and physical activity levels, and lower binge eating; no effect of age or illness duration	2
Nisticò et al. (2021)	Italy	40 (95%)	30.9 (14.2)	100% White	Not reported	Not reported	AN *n* = 15; BN *n =* 11; BED *n =* 14	Longitudinal	ED examination; depression, anxiety, stress; psychological distress	Posttraumatic stress (IES‐R total score: *η* ^2^_p_ = 0.145) and ED symptoms (*η* ^2^_p_ = 0.142–0.249) improved from during to postlockdown; no change in stress, anxiety, or depression	2
Pfund et al. (2020)	United States	438 (gender identity: 100% women; 434 ciswomen and 4 transwomen)	31.3 (12.7)	11% African American/Black; 22% Asian/Asian American; 52% European American/White; 10% Latinx American/Hispanic; 2% Middle Eastern, and 3% “other”/not disclosed	Not reported	Not reported	n/a	Cross‐sectional	Body surveillance; appearance comparison; body satisfaction	Time video chatting increased from pre‐ to post‐COVID (*d* = 0.53). Time video chatting not associated with appearance satisfaction; self‐objectification moderated relationship between time video chatting and appearance satisfaction (*B* = −.04 for face satisfaction and *B* = −.02 for body satisfaction); participants who spent more time engaged with their families over video chatting services reported greater face (*r* = .21) and body (*r* = .17) satisfaction	2
Phelan et al. (2021)	Ireland	1031 (sex assigned at birth: 100% female)	36.7 (6.6)	97% White; 2% Asian; 0.3% Black; 0.5% “other”	Not reported	Not reported	n/a	Cross‐sectional	Mental health symptoms, diet, exercise	Participants reported an increase in binge eating from pre‐ to during COVID	3
Philippe et al. (2021)	France	498 (52%)	7.3 (2.3)	Not reported	Not reported	Not reported	n/a	Cross‐sectional (retrospective)	Eating difficulties; eating behavior; parental feeding practices	Parents reported an increase in their child's emotional overeating, food responsiveness, food enjoyment, and appetite, but no change in their child's pickiness from pre‐ to during lockdown; boredom predicted increased food responsiveness (*β* = .14), emotional overeating (*β* = .20), and snack frequency between meals (*β* = .28)	2
Phillipou et al. (2020)	Australia	5469 (96% women; 3% preferred to self‐describe)	30.5 (8.2)	Not reported	Not reported	Not reported	AN *n* = 88; BN *n* = 23; BED *n* = 6; OSFED *n =* 4; UFED *n* = 68; recovering/in recovery *n* = 10	Cross‐sectional	Depression, anxiety, stress; ED examination	Participants with ED history reported increased restricting (64.5%), binge eating (35.5%), purging (18.9%), and exercise behaviors (47.3%); participants without ED history reported both increased restricting (27.6%) and binge eating behaviors (34.6%), but decreased exercise (43.4%) from pre‐ to during COVID	3
Pikoos et al. (2020)	Australia	216 (gender: 88% women; 0.01% “other”)	32.5 (11.8)	Not reported	Not reported	Not reported	n/a	Cross‐sectional	Dysmorphic concern; depression, anxiety, stress; appearance‐focused behaviors	Appearance‐focused behaviors decreased in participants with low dysmorphic concerns, but not in participants with high dysmorphic concerns from pre‐ to during COVID; living alone, younger age, higher dysmorphic concern, and greater distress over beauty service closure predicted appearance‐focused behaviors	2
Puhl et al. (2020)	United States	584 (gender identity: 64% women; 1% “other”)	24.6 (2.0)	30.2% White; 16.8% African American/ Black; 17.1% Hispanic; 24.3% Asian American; 11.6% “other”	Not reported	31.5% lower class; 20.0% lower middle class; 17.4% middle class; 19.4% upper middle class; 11.7% upper class (assumed self‐report)	n/a	Longitudinal	Psychological distress; eating behaviors; binge eating; physical activity; weight stigma	Pre‐COVID experiences of weight stigma predicted higher levels of depressive symptoms (*β* = .15), stress (*β* = .15), eating as a coping strategy (*β* = .16), and an increased likelihood of binge eating during COVID (OR = 2.88), but were unrelated to physical activity; no effect of gender	2
Ramalho et al. (2021)	Portugal	254 (83%)	35.8 (11.8)	Not reported	Not reported	Education: 13.0% high school; 27.2% bachelor's degree; 59.8% master's degree/doctorate	n/a	Cross‐sectional	DE behaviors; COVID impact; depression, anxiety, stress; ED symptoms	Psychosocial impact of COVID predicted DE behaviors mediated through psychological distress (>women; younger age) (*β* = .10); psychosocial impact of COVID associated with emotional eating (*r* = .23) and uncontrolled eating (*r* = .18)	2
Richardson et al. (2020)^b^	Canada	439 (gender: 80% women; 2% transgender; 11% did not disclose)	Not reported	Not reported	Not reported	Not reported	AN *n* = 83; BN *n* = 44; ARFID *n =* 4; BED *n* = 66; OSFED *n =* 9; undisclosed/ undiagnosed *n* = 233	Cross‐sectional (retrospective)	Telephone survey	Service utilization, ED symptoms, anxiety, and depression increased from pre‐ to during COVID among patients with EDs	3
Robertson et al. (2021)	United Kingdom	264 (78%)	Range 18–79 years	92% White	Not reported	Not reported	13.8% current or past ED diagnosis	Cross‐sectional	Perceived change in eating, exercise, and BI; patient health	53% reported more difficulty regulating eating; 60% reported more preoccupation with food/eating; 50% reported exercising more; 68% reported thinking more about exercise; 49% reported more appearance concerns from pre‐ to during lockdown (>women; participants with past/current ED); psychological distress was correlated with finding it more difficult to control/regulate one's eating (*r*s = .36), being more preoccupied with food/eating (*r*s = .29), thinking more about exercise (*r*s = .17), and being more concerned about one's appearance (*r*s = .41)	3
Scharmer et al. (2020)	United States	295 (65%)	19.7 (2.0)	48% White; 21% African American; 11% Asian; 14% Hispanic/Latino; 1% Native American; 1% Native Hawaiian/Pacific Islander; 3% “other”	Not reported	Not reported	n/a	Cross‐sectional	ED examination; compulsive exercise; anxiety; fear of illness and virus evaluation; intolerance of uncertainty; physical activity	COVID anxiety and intolerance of uncertainty was associated with ED pathology, but not compulsive exercise; trait and COVID intolerance of uncertainty moderated associations between COVID anxiety and compulsive exercise and ED pathology; COVID anxiety was more strongly related to compulsive exercise and ED pathology for individuals with lower intolerance of uncertainty	3
Schlegl, Maier, et al. (2020)	Germany	159 (100%)	22.4 (8.7)	Not reported	Not reported	Not reported	AN	Cross‐sectional (retrospective)	Psychological consequences of COVID	>70% reported that eating, shape and weight concerns, drive for physical activity, loneliness, sadness, and inner restlessness increased from pre‐ to during COVID and access to in person care decreased; participants reported daily routines, day planning, and enjoyable activities as the most helpful coping strategies; reduction in overall ED symptoms/taking on responsibility to recover, reduction in specific ED symptoms, more flexibility regarding meals and foods, wake‐up call/will to live, trying out therapy content, and accepting uncertainty in life were reported as positive impacts of COVID	3
Schlegl, Meule, et al. (2020)	Germany	55 (100%)	24.4 (6.4)	Not reported	Not reported	Not reported	BN	Cross‐sectional (retrospective)	Psychological consequences of COVID	49% reported deterioration of ED symptomatology and 62% reported reduced quality of life; frequency of binge eating increased in 47% of patients, self‐induced vomiting in 36%, laxative use in 9%, and diuretic abuse in 7%; face‐to‐face psychotherapy decreased by 56%, videoconferencing therapy was used by 22% of patients; enjoyable activities, virtual contact with friends, and mild physical activity rated as most helpful coping strategies	3
Serin and Koç (2020)	Turkey	1064 (59%)	Not reported	Not reported	Not reported	Not reported	n/a	Cross‐sectional	Eating behaviors; depression	External eating, but not emotional or restrictive eating, higher in participants who reported self‐isolating, compared to those who did not (>women)	2
Spettigue et al. (2021)	Canada	48 (gender: 83% ciswomen; 2% transwomen; 4% transmen)	14.6 (1.8)	Not reported	Not reported	Not reported	AN‐R *n* = 24; AN‐BP *n* = 7; ARFID *n* = 7; AAN *n* = 6; BN *n* = 1; UFED *n* = 3	Cohort	ED examination; eating attitudes; clinical impairment	40% cited pandemic as trigger for ED; inpatient admissions, emergency room consultation requests, and outpatient referrals deemed “urgent” were higher during COVID compared to pre‐COVID; compared to 2019 ED patients, ED patients in 2020 reported worse clinical impairment from ED symptoms (*d* = 0.44) and higher levels of eating restraint (*d* = 0.63)	2
Stoddard (2021)^b^	United States	69 (gender identity: 96% women; 4% nonbinary)	96% 18–34 years	91% White; 7% Black; 3% Asian; 6% Latina; 3% “other”	Not reported	Not reported	Previous/current: 83/71% BI disturbance; 84/51% DE habits; 72/13% ED	Cross‐sectional	ED/BI status/concern level; impact of COVID; experiences of ED/BI; coping mechanisms	84% reported concern about how their weight/bodies might be affected by COVID; 59% reported that their BI/ED worsened from pre‐ to post‐COVID; 78% reported that their relationships with their bodies have changed (70% negative, 26% positive, 4% neutral)	2
Swami, Horne, et al. (2021)	United Kingdom	506 (gender identity: 44% women)	34.3 (11.4)	88.5% White	89.1% heterosexual	Education: 10.9% high school; 27.9% advanced qualification; 38.3% undergraduate degree; 19.0% postgraduate degree; 3.9% other	n/a	Cross‐sectional	Perceived stress; ED inventory; body attitude; COVID‐related stress and anxiety	In women, COVID‐related anxiety associated with body dissatisfaction and COVID‐related anxiety and stress was associated with drive for thinness; in men, COVID‐related anxiety was associated with low body fat dissatisfaction and COVID‐related anxiety and stress was associated with muscularity dissatisfaction	2
Swami, Todd, et al. (2021)	United Kingdom	600 (gender identity: 49% women)	34.6 (12.3)	85% White; 9% Asian; 2% Black; 4% mixed race; 0.3% “other”	87% heterosexual; 3% gay/lesbian; 8% bisexual; 2% identified with another orientation	Not reported	n/a	Cross‐sectional	BI disturbance; COVID‐related stress; self‐compassion	COVID‐related stress associated with greater BI disturbance, mediated by lower self‐compassion; self‐compassion did not moderate effects of stress on BI disturbance	2
Tabler et al. (2021)^b^	United States	411 (74% women; 5% transgender, genderqueer, or nonbinary)	28.5 (11.4)	86% White; 14% Latinx	71% heterosexual; 29% lesbian, gay, bisexual, or queer	44% working class; 44% middle class; 12% upper middle class (self‐report)	n/a	Cross‐sectional	ED examination; pandemic‐related stress	Pandemic‐related stress associated with ED symptoms and perceived weight gain (>LGBTQ+ individuals)	2
Taquet et al. (2021)	United States	5,186,451 (55%)	15.4 (9.0)	Not reported	Not reported	Not reported	Patients with any ED diagnosis	Cross‐sectional (retrospective)	Incidence of ED diagnosis	Diagnostic incidence of EDs 15.3% higher in 2020 compared with previous 3 years; increase occurred solely in women, and primarily related to teenagers and AN; higher proportion of patients with EDs in 2020 had suicidal ideation or attempted suicide	1
Termorshuizen et al. (2020)	United States; Netherlands	United States/Netherland: 511/510 (gender identity: 95/98% women; 2/0.6% nonbinary/genderfluid/“other”)	United States: 30.6 (9.4); Netherlands: 90% 16–39 years	Not reported	Not reported	Not reported	AN *n* = 665; BN *n* = 295; BED *n* = 216; AAN *n* = 203; OSFED *n* = 192; purging disorder *n* = 47; ARFID *n* = 36; night‐eating syndrome *n* = 25	Cross‐sectional	Impact of COVID on EDs, general physical and mental well‐being, and ED treatment	Participants with AN reported increased restriction and fears about being able to find foods consistent with meal plan from pre‐ to during COVID; participants with BN and BED reported increases in binge eating and urges to binge; participants reported positive effects of COVID including greater connection with family, more time for self‐care, and motivation to recover	2
Trott et al. (2021)	United Kingdom	319 (84%)	36.7 (11.8)	Not reported	Not reported	Not reported	n/a	Longitudinal	Body dysmorphic symptoms; eating attitudes; exercise addiction	ED symptomatology and exercise increased while exercise addiction decreased from pre to postlockdown; no changes in body dysmorphic symptoms from pre‐ to postlockdown	2
Vall‐Roqué et al. (2021)	Spain	2601 (gender: 100% women)	24.1 (5.0)	Not reported	Not reported	Not reported	n/a	Cross‐sectional (retrospective)	ED inventory; social network sites use; self‐esteem	Social media network site use increased from pre‐ to during lockdown and was associated with lower self‐esteem (*g* = 0.15 for 14–24 year olds), higher body dissatisfaction (*g* = −0.14 for 14–24 year olds), and higher drive for thinness (*g* = −0.18 for 14–24 year olds, *g* = −0.22 for 25–35 year olds) (>younger age)	2
Vitagliano et al. (2021)	United States	89 (sex assigned at birth: 89% female)	18.9 (2.9)	78% White, non‐Hispanic; 8% Asian; 7% Multiracial; 4% Hispanic; 1% Black; 2% “other”	Not reported	Not reported	84% restrictive ED diagnosis; 16% other ED diagnosis	Cross‐sectional	ED related concerns and motivation to recover; triggering environment; ED diagnosis	63% reported concern for worsening of their ED due to a “triggering environment”; 74% reported an increase in ED thoughts, 77% reported anxiety, 73% reported depression, and 80% reported isolation they perceived to be related to COVID; 29% reported decrease in motivation to recover they perceived to be related to COVID; participants who reported concern for worsening of their ED due to a triggering environment expressed decreased motivation to recover (OR = 18.1) and increased ED thoughts (OR = 23.8) compared to those who did not report concern for worsening of their ED due to a triggering environment	3
Vuillier et al. (2021)^b^	United Kingdom	207 (63%)	30.0 (9.7)	93.7% White British/Irish/Scottish/European; 5.3% Asian; 0.5% Black; 0.5% Arab	Not reported	Not reported	AN *n* = 91; BN *n* = 46; BED *n =* 44; OSFED *n* = 26	Cross‐sectional	Depression, anxiety, stress; ED examination	83.1% reported worsening of ED symptomatology, 7.7% reported no change, 6.8% reported improvements in ED symptomatology; changes did not differ based on diagnosis; changes to routine and physical activity and emotion difficulties were most important factors in predicting change in ED symptoms	3
White III (2021)	United States	311 (70%)	Not reported	33.1% African American; 55.3% White; 11.6% “other”	Not reported	Not reported	n/a	Cross‐sectional (retrospective)	Physical activity; self‐esteem; BI	Self‐esteem and BI worsened from pre‐ to during COVID	3
Zhou and Wade (2021)	Australia	100 (100%)	19.9 (2.0)	88% White; 6% Asian; 6% “other”	Not reported	Not reported	n/a	Cohort (part of randomized controlled trial)	ED examination; BI acceptance; self‐compassion	Weight concerns (*d* = 0.46), DE (fasting, binge eating, vomiting, and driven exercise; *d* = 0.55), and negative affect (*d* = 0.40) increased from pre‐ to during COVID, all associated with moderate effect sizes	2

*Note*: Study quality was assessed using the EPHPP guidelines as follows: 1 = “strong,” 2 = “moderate,” 3 = “weak.” Effect size interpretation: Cohen's *d*/Hedge's *g*: 0.2 = small, 0.5 = medium, 0.8 = large; *η* ^2^/*η* ^2^_p_: 0.01 = small, 0.06 = medium, 0.14 = large; *r* ^2^: 0.01 = small, 0.09 = medium, 0.25 = large; odds ratio (OR): 1.68 = small, 3.47 = medium, 6.71 = large^c^; Pearson's *r*: 0.1 = small, 0.3 = medium, 0.5 = large.

Abbreviations: AN, anorexia nervosa; AAN, atypical anorexia; AN‐BP, binging/purging anorexia subtype; AN‐R, restrictive anorexia subtype; ARFID, avoidant restrictive food intake disorder; BED, binge eating disorder; BI, body image; BN, bulimia nervosa; DE, disordered eating; ED, eating disorder; OSFED, other specified feeding or eating disorder; UFED, unspecified feeding or eating disorder.

^a^ The majority of included studies did not specify whether they assessed sex assigned at birth, gender, or gender identity; where this information is available, sex assigned at birth, gender, and gender identity are reported separately.

^b^ Signifies a multimethods paper. Information detailed here concerns the quantitative methods, analysis, and findings. See Table 2 for the qualitative characteristics of this study.

^c^ Chen, H., Cohen, P., & Chen, S. (2010). How big is a big odds ratio? Interpreting the magnitudes of odds ratios in epidemiological studies. *Communications in Statistics—Simulation and Computation*, *39*(4), 860–864.

**TABLE 2:** Characteristics of qualitative studies

Author(s) (year)	Country	Participants						Methodology		Key findings	Study quality
		*N* (% female)^a^	Age *M* (*SD*)	Race and ethnicity	Sexual orientation	Socioeconomic status	Diagnosis	Data collection methods	Data analysis		
Branley‐Bell and Talbot (2020)^b^	United Kingdom	129 (94%)	29.3 (89.0)	Not reported	Not reported	Not reported	62% current ED/relapse; in recovery 6.2% <3 m, 6.2% 3–12 m, 25.6% >12 m	Online survey	Thematic analysis	Themes generated: (1) disruption to living situation; (2) increased social isolation and reduced access to usual support networks; (3) changes to physical activity rates; (4) reduced access to healthcare services; (5) disruption to routine and perceived control; (6) increased exposure to triggering messages; (7) changes to the individual's relationship with food; (8) positive outcomes	6.5
Branley‐Bell and Talbot (2021)^b^	United Kingdom	58 (98%)	30.9 (11.1)	Not reported	Not reported	Not reported	63.8% current ED/relapse; 36.2% in recovery; AN *n* = 28; BN *n =* 7; OSFED *n =* 3; BED *n* = 2; symptoms of multiple EDs *n* = 12; undisclosed ED *n* = 7	Online survey	Thematic analysis	Themes generated: (1) ED behaviors as an ‘auxiliary control mechanism’; (2) loss of auxiliary control after lockdown	6.5
Brown et al. (2021)	United Kingdom	10 (90% women; 10% nonbinary)	29.5 (5.0)	100% White	Not reported	Not reported	OSFED *n* = 2; AN *n* = 6; BED *n* = 1; “other” *n* = 1	Semi‐structured interviews	Thematic analysis	Themes generated: (1) social restrictions; (2) functional restrictions; and (3) restrictions in access to mental health services	10
Buckley et al. (2021)^b^	Multiple	204 (86%)	27.0 (8.1)	Not reported	Not reported	Not reported	10.7% current ED diagnosis; 32.8% previous ED diagnosis (AN *n* = 29; BN *n* = 11; ON *n* = 9; BED *n* = 7; “other” *n* = 11)	Online survey	Content analysis	Content analysis highlighted worsened BI, worsened relationship with food, and additional COVID challenges; DE occurred predominantly in the form of binge eating, body preoccupation, fear of body composition changes, and inhibitory food control	7.5
Clark Bryan et al. (2020)	United Kingdom	21 (86%)	25.5 (5.6)	Not reported	Not reported	Not reported	AN	Semi‐structured interviews	Thematic analysis	Themes generated: (1) reduced access to ED services; (2) disruption to routine and activities in the community; (3) heightened psychological distress and ED symptoms; and (4) increased attempts at self‐management in recovery	6
Fernández‐Aranda, Casas, et al. (2020) Study 2	Spain	8 (not reported)	Not reported	Not reported	Not reported	Not reported	AN	Text‐based focus group	Thematic analysis	Themes generated: (1) connecting in isolation; (2) helping others versus helping oneself; (3) challenges of reduced professional support; (4) balancing the needs of the individual within the family	3
Frayn et al. (2021)	United States	11 (64% women; 9% transmen)	42.8 (14.2)	81.8% White; 18.2% Black	Not reported	Household annual income (USD): 9.1% 0–10,000; 9.1% 25,001–30,000; 9.1% 30,001–35,000; 9.1% 45,001–50,000; 9.1% 70,001–75,000; 45.5% 100,000+	BED *n* = 7; BN *n* = 2; OSFED *n* = 2	Semi‐structured interviews	Thematic analysis	Themes generated: (1) variability in improvement or exacerbation of symptoms due to COVID; (2) changes in the physical environment were associated with symptom improvement; (3) social implications of COVID associated with both symptom improvement and deterioration; (4) greater overall stress/anxiety levels led to more binge episodes	7.5
Hunter and Gibson et al. (2021)	United States; Greece; United Kingdom	12 (92%)	31.8 (not reported)	Not reported	Not reported	Not reported	AN *n =* 10; AN and BN *n* = 1; BN *n* = 1	Semi‐structured interviews	Thematic analysis	Themes generated: (1) loss of control; (2) support during confinement; (3) time of reflection on recovery	8
McCombie et al. (2020)	United Kingdom	32 (94%)	35.2 (10.3)	100% White	Not reported	Not reported	AN *n =* 23; BN *n* = 3; BED *n* = 1; “other” *n* = 5	Online survey	Thematic analysis	Themes generated: (1) mechanisms contributing to ED exacerbation; (2) positive aspects of lockdown	7.5
Nutley et al. (2021)	Multiple	305 social media posts	Not reported	Not reported	Not reported	Not reported	Not reported	Subreddit posts (r/EatingDisorders, r/AnorexiaNervosa, r/BingeEatingDisorder	Thematic analysis	Themes generated: (1) change in ED symptoms; (2) change in exercise routine; (3) impact of quarantine on daily life; (4) emotional well‐being; (5) help‐seeking behavior; (6) associated risks and health outcomes	7.5
Quathamer and Joy (2021)	Canada	Survey: 70 (gender: 45% women; 22% agendered, nonbinary, genderfluid, or gender vague; 9% transgender); interview: 8 (gender: 50% ciswomen; 12.5% transwomen; 25% nonbinary)	Not reported	Survey: 81% White; 9% Indigenous; 6% Latinx; 3% Asian; 1% Middle Eastern; interview: 87.5% White; 12.5% South Asian	Survey: 27% gay; 23% bisexual; 17% lesbian; 12% queer; 6% pansexual; 3% 2‐spirit; 3% demisexual; 1% nonfixed; 1% questioning; interview: 12.5% gay; 25% lesbian; 25% bisexual; 12.5% queer; 12.5% asexual; 12.5% nonspecified	Not reported	n/a	Online survey and semi‐structured interviews	Foucauldian discourse analysis	Discursive considerations generated: (1) time for reflection; (2) time away from social surveillance; (3) time to work on oneself; and (4) time to (dis)connect; woven through these considerations were social discourses of hetero‐cis‐normativity, healthism, and resistance	10
Richardson et al. (2020)^b^	Canada	56 chat transcripts	Not reported	Not reported	Not reported	Not reported	Not reported	Textual analysis of chat transcripts	Thematic analysis	Themes generated: (1) lack of access to treatment; (2) worsening of symptoms; (3) feeling out of control; (4) need for support	8
Simone et al. (2021)	United States	510 (not reported)	24.7 (2.0)	29.6% White; 23.9% Asian; 16.5% Latino/Hispanic; 18.2% African American/Black; 11.8% mixed/“other”	Not reported	32.7% low; 20.7% low‐middle; 17.0% middle; 18.5% upper‐middle; 11.2% high (parental socioeconomic status primarily based on baseline educational attainment)	n/a	Online survey	Thematic analysis	Themes generated: (1) mindless eating and snacking; (2) increased food consumption; (3) generalized decrease in appetite or dietary intake; (4) eating to cope; (5) pandemic‐related reductions in dietary intake; (6) re‐emergence or marked increase in ED symptoms	8
Stoddard (2021)^b^	United States	17 (gender identity: 100% women)	Not reported	Not reported	Not reported	Not reported	Not reported	Semi‐structured interviews and autoethnography	Content analysis	Themes generated: (1) influencing factors for BI disturbance/EDs during COVID (stress and distress; social isolation; time; activity level changes; media; control; external situations; other mental health issues; weight change); (2) coping mechanisms; (3) hopes and fears	9.5
Tabler et al. (2021)^b^	United States	43 (63% ciswomen; 2% transwomen; 7% nonbinary; 5% queer)	27.7 (9.2)	79% White; 7% biracial/multiracial; 12% Latinx/Hispanic; 2% Asian American	19% lesbian; 16% gay; 35% bisexual; 7% queer; 12% pansexual; 5% asexual; 7% expansive sexuality/unlabeled	44% working class; 44% middle class; 12% upper middle class (self‐report)	n/a	Semi‐structured interviews	Thematic analysis	Themes generated: (1) physical activity constraints; (2) eating patterns; (3) weight concerns	7
Vuillier et al. (2021)^b^	United Kingdom	207 (63%)	30.0 (9.7)	93.7% White British/Irish/Scottish/European; 5.3% Asian; 0.5% Black; 0.5% Arab	Not reported	Not reported	AN *n* = 91; BN *n* = 46; BED *n =* 44; OSFED *n* = 26	Online survey	Thematic Analysis	Themes generated regarding impact of COVID: (1) difficult emotions; (2) changes to routine; (3) confinement; (4) unhelpful social messages—making a transformation; (5) emotional support from others; (6) emotion coping skills; (7) accessibility—barriers and facilitators; (8) loss of support	7
Zeiler et al. (2021)	Austria	13 (100%)	15.9 (1.4)	Not reported	Not reported	Not reported	AN‐R *n* = 9; AN‐BP *n* = 4	Semi‐structured interviews	Thematic analysis	Themes generated: (1) restrictions of personal freedom; (2) interruption of treatment routine; (3) changes in psychopathology; (4) opportunities of COVID period	7.5

*Note*: Study quality was assessed using an adapted version of the critical appraisal skills program (CASP) tool (Long et al., 2020).

Abbreviations: AAN, atypical anorexia; AN, anorexia nervosa; AN‐BP, binging/purging anorexia subtype; AN‐R, restrictive anorexia subtype; ARFID, avoidant restrictive food intake disorder; BED, binge eating disorder; BI, body image; BN, bulimia nervosa; DE, disordered eating; ED, eating disorder; OSFED, other specified feeding or eating disorder; UFED, unspecified feeding or eating disorder.

^a^ The majority of included studies did not specify whether they assessed sex assigned at birth, gender, or gender identity; where this information is available, sex assigned at birth, gender, and gender identity are reported separately.

^b^ Signifies a multimethods paper. Information detailed here concerns the qualitative methods, analysis, and findings. See Table 1 for the quantitative characteristics of this study.

### 2.5 Synthesis of results

Due to the inclusion of mixed‐methods studies and the heterogeneity of existing evidence, we adopted a narrative synthesis approach informed by the guidance by Popay et al. (2006): (1) developing a theory; (2) developing a preliminary synthesis; (3) exploring relationships in the data; and (4) assessing robustness of the synthesis.

#### 2.5.1 Stage 1: Development of theory

This stage was performed early on in the review process and helped shape the review aims. Through an initial review of the literature and discussion among the research team, we identified several possible mechanisms whereby the COVID‐19 pandemic and related factors may influence BI, DE, and EDs, such as psychological distress due to the ongoing pandemic, disruption of access to support and treatment, food shortage and insecurity, and increased loneliness and social isolation. Key findings from the literature review are outlined in the introduction section of this manuscript. This process also highlighted a lack of studies focusing on individuals from marginalized and underrepresented populations, which we included as a key aim of our review.

#### 2.5.2 Stage 2: Development of the preliminary synthesis

The second stage involved organizing and describing the included papers to explore patterns across studies. We followed Stern et al.'s (2020) guidance on conducting mixed‐studies systematic reviews, as outlined in the Joanna Briggs Institute (JBI) manual for evidence synthesis. Accordingly, we conducted the current review using the convergent integrated approach (Hong et al., 2017), whereby findings from the qualitative, quantitative, and mixed‐methods studies were integrated in the narrative synthesis. Quantitative data were transformed (or *qualitized*) into “textual descriptions” and presented in conjunction with qualitative data. This approach is recommended over its counterpart where qualitative data are assigned numerical values (*quantitized*), as codifying quantitative data is less error‐prone than attributing numerical values to qualitative data. One of the distinguishing features of mixed‐studies systematic reviews is the inclusion of primary mixed‐methods studies, from which data are extracted so they can be classified as quantitative or qualitative (Stern et al., 2020). Therefore, quantitative data from mixed‐methods studies were also transformed and synthesized with quantitative data from quantitative studies and nontransformed qualitative data from qualitative and mixed‐methods studies. Due to the variability in the emerging literature and the need to comprehensively capture all relevant findings, the themes and resulting narrative synthesis were based on a comprehensive examination of all included papers, regardless of study quality.

#### 2.5.3 Stage 3: Exploring the relationships within and between studies

In this stage, four main themes (and two subthemes) were generated from the integrated qualitative and transformed quantitative data to answer the key research aims. The themes were generated using deductive, or theory‐driven approaches (i.e., considering data relevant to answering the review questions) and inductive, or data‐driven approaches (i.e., being open to the data that are present across studies and unexpected findings; Clarke & Braun, 2014). All themes were informed by *both* qualitative and quantitative research. For clarity, a selection of references is provided in the narrative synthesis; please see Tables 1 and 2 for a detailed breakdown of study findings and associated citations. Illustrative quotes for each theme are presented in Table 3.

**TABLE 3:** Participant quotes

Theme		Quote	Author
1. Disruptions due to COVID‐19		“Feeling frustrated because I've been learning how to control the bulimic symptoms and normally able to manage them. Yet the change in routine (or lack of) and general stress/anxiety is unsettling it and I noticed my thoughts and behaviors changing” *(participant details not provided)*	Branley‐Bell and Talbot (2020)
		“Without structure and focus (and meaning) of uni [university] and friends and having a life that feels worth living, now it's just old habits and misery” *(participant details not provided)*	McCombie et al. (2020)
		“My routine has gotten lost, which tampers with my sleep, which in turn tampers with my ability to follow my meal plan” *(30, female participant, anorexia nervosa)*	Hunter and Gibson (2021)
		“For me, it was like, I go to my therapist, I leave my problems there, I go home. […] Now, many things take place in my room, and I have all these problems, these conversations right on the spot. This felt strange for me at the beginning” *(participant details not provided)*	Zeiler et al. (2021)
2. Variability in the improvement or exacerbation of symptoms	2.1 Negative outcomes of the COVID‐19 pandemic	“I have been in recovery from AN [anorexia nervosa] and BN [bulimia nervosa] for about 2 years now, and ever since this whole isolation thing started, my symptoms have come back, and worsened almost immediately” *(participant details not provided)*	Richardson et al. (2020)
		“The pandemic has increased my self‐harm, as I've felt so restricted by the government in terms of what I can do to deal with my feelings (i.e., initially – only exercise once a day, not able to exercise with anyone else). Using healthy means to deal with difficult feelings (i.e., go for a walk, meet a friend for coffee) have been more limited and so it is really easy to go back to unhelpful ways of coping such as self‐harm” *(female participant, anorexia nervosa)*	Vuillier et al. (2021)
		“My ED feels more valuable to me than ever. It is the only constant in what feels like a completely upside down and scary world, it is my only locus of control” *(participant details not provided)*	Branley‐Bell and Talbot (2021)
		“With the recent quarantine, I am unable to work out the same… just running and doing as much as I can. I've found my body changing in ways I am very uncomfortable with. I'm waking up daily weighing myself, logging my food, and checking my Fitbit constantly. I recently started staring in the mirror more and despising the person who looks back” *(participant details not provided)*	Nutley et al. (2021)
		“Times when I would normally kind of be doing something potentially social or something like that over the weekend… Obviously with more free time, I might have gone back to see my parents–that […] feeling, of like, existential loneliness felt incredibly desperate and really quite painful. But it was… It came in bursts to begin with, and I think as lockdown has gone on, it's that feeling of real painful loneliness” *(25, female participant, White, anorexia nervosa)*	Brown et al. (2021)
		“Everything is dramatically worse because of being home all the time. I have constant access to all of my food and my scale and everything to evaluate where I'm at and to facilitate eating” *(participant details not provided)*	Frayn et al. (2021)
		“I definitely walk past the mirror more often and do some body checking” *(participant details not provided)*	McCombie et al. (2020)
		“In the initial stages of the pandemic it seemed pointless to try to manage my ED [eating disorder]. The stress and anxiety regarding the future meant that I did not see the point in investing energy in my own physical or mental health” *(female participant, binge eating disorder)*	Vuillier et al. (2021)
		“It's sort of paradoxical because on one hand, I was really anxious realizing it was going to stall my progress but then on the other, I was really anxious that it would stall my disordered behaviors, so it's been quite complex” *(27, female participant, anorexia nervosa)*	Hunter and Gibson (2021)
		“COVID‐19 has affected my general mental health quite a lot through isolation and anxiety. I think this is having as much of an effect on my fitness and relationship with my body as anything else” *(participant details not provided)*	Buckley et al. (2021)
	2.2 Positive outcomes of the COVID‐19 pandemic	“Binging […] and purging has reduced. I was doing this five times a day. Now people are home with me my binge purge has reduced to every other day” *(participant details not provided)*	Branley‐Bell and Talbot (2020)
		“Your immune system is really weakened because of an ED [eating disorder] and for me, I do not want that anymore so I'm feeling really motivated to kind of improve my physical health as much as possible” *(participant details not provided)*	Clark Bryan et al. (2020)
		“But I very quickly was able to turn it around and make it an opportunity for healing and like, I've dedicated so much time to eating disorder recovery during quarantine that I felt like that's what it was for. […] And so, I literally like am living in this renaissance of intuitive eating and of like, anti‐diet‐culture activism, because I found so much space for it during quarantine” *(female participant)*	Stoddard et al. (2021)
		“Having the space to do that [reflect on recovery] is really good but I definitely did think that I was further along in my recovery than I am, but I guess that is a good thing because it means yeah I guess it is a good thing because it means I am aware of it as well which is helpful” *(29, female participant, anorexia nervosa)*	Hunter and Gibson (2021)
3. Factors associated with body image and disordered eating outcomes		“I'm furloughed so have had the mental space to acknowledge that I do actually have a problem. I have started recognizing my own ED [eating disorder] thought patterns and behaviors and am working on challenging them” *(participant details not provided)*	McCombie et al. (2020)
		“My symptoms have probably gotten better because I am home and cannot go anywhere. Before my binge episodes were on the road and I was by myself. The fact that I am physically constrained and do not have access to what I had before helps. I do not go out much, not by myself. I am with my kids and family, so I do not have privacy and am very conscious of that” *(participant details not provided)*	Frayn et al. (2021)
		“I am also a lot more honest with my family and friends about the struggles I face so I have constant support from them when I need it” *(female participant, anorexia nervosa)*	Vuillier et al. (2021)
		“[Messages were] about not ‘letting oneself go’ or how terrible it would be to gain weight in quarantine, or how people were supposed to work hard to somehow come out of this ‘better than ever’, and boy, did the ED [eating disorder] voice in me love that” *(White, bisexual, genderfluid participant)*	Quathamer and Joy (2021)
		“COVID‐19 has brought back many of the habits I had when I was struggling with my ED [eating disorder]. The spread of the jokes about the ‘corona 15′ were what sparked a lot of the unhealthy habits resurfacing” *(22, female participant, White)*	Simone et al. (2021)
		“My mom had always joked about my body/weight (since I used to be overweight, she would joke about me not being able to fit through my door). She does not mean any harm, but I get really devastated when she mentioned that I've gained weight during quarantine. I started to restrict even more” *(participant details not provided)*	Nutley et al. (2021)
		“I'm trapped at home with people who do not know I have anorexia. I am hiding and lying constantly” *(participant details not provided)*	Branley‐Bell and Talbot (2020)
		It has meant that I have no one around to keep an eye on me so I can binge when I want to and then make myself sick to make myself feel better and I do not have to explain it to anyone because there is no one there to hear me” *(male participant, bulimia nervosa)*	Vuillier et al. (2021)
		“It's a very secretive disorder and if I'm face to face with someone [for treatment] I cannot lie, I cannot hide the fact that I've lost weight or hide the fact that I'm drained energy‐wise, whereas on the phone I can cover up” *(29, female participant, anorexia nervosa)*	Hunter and Gibson (2021)
4. Unique challenges for marginalized and underrepresented groups		“I was always concerned about how I look externally but ever since the pandemic the focus has been so much more because the way I justify it is I have more time to work on my body” *(South Asian, gay, cisgender man)*	Quathamer and Joy (2021)
		“I found myself snacking more than usual. Some days I would overeat, other days I would barely eat anything. The major concern has always been my weight. So that's something I'm looking forward to, like getting back on track with the whole, exercising, and trying not to snack as much as before” *(Latina, pansexual, cisgender woman)*	Tabler et al. (2021)
		“I relished the opportunity to be comfortable at home so much and to not have to feel like I was presenting my body and gender in a socially acceptable way. I loved eating things that gave me pleasure without fear of outside judgment and connecting with my body through exercise in a slow and easy way” *(White, bisexual, genderfluid participant)*	Quathamer and Joy (2021)
		“The pandemic made me stay with my unaccepting parents who do not respect my identity and limited my presentation to ways that made me feel very dysphoric and negative about my body” *(White, lesbian, transgender woman)*	Quathamer and Joy (2021)

#### 2.5.4 Stage 4: Evaluating the robustness of the synthesis

In the final stage, the methodological quality of the included studies and of the review process was examined to assess the strength of the evidence presented within the review. We provide considerations and implications of the review's findings in the discussion section of this manuscript.

### 2.6 Quality assessment

Due to the novelty of COVID‐19 and the need to gain a comprehensive understanding of its impact on BI and DE, no studies were excluded as a result of the quality assessments. Rather, quality scores of the included studies are provided in Tables 1 and 2 and considered in the results and discussion sections of this manuscript.

#### 2.6.1 Quantitative studies

Quality of quantitative studies was assessed using the Quality Assessment Tool for Quantitative Studies, developed by the Effective Public Health Practice Project (EPHPP; Thomas et al., 2004). The EPHPP provides an overall methodological quality rating of “strong” (no weak component ratings), “moderate” (one weak component rating), or “weak” (two or more weak component ratings). The ratings are based on the following components: selection bias, study design, confounders, blinding, data collection method, and withdrawals and dropouts. The EPHPP is suitable for evaluating the methodological quality of various study designs (Jackson & Waters, 2005). In addition, the EPHPP has excellent interrater reliability for overall scores when compared to the Cochrane Collaboration Risk of Bias Tool (Armijo‐Olivo et al., 2012) and established construct and content validity (Jackson & Waters, 2005). Two authors (B.G. and D.T.) independently assessed all studies. Cohen's kappa (Cohen, 1960) was calculated to determine interrater reliability, showing good agreement (92%) between total scores (*κ* = 0.866, *p* < .001). Discrepancies were resolved by a third author (J.S.), who rated all papers in line with either BG or DT, and the majority score was assigned.

#### 2.6.2 Qualitative studies

Quality of qualitative studies was assessed using a modified version of the 10‐item Critical Appraisal Skills Program (CASP) qualitative checklist, as detailed by Long et al. (2020). The CASP checklist is the most commonly used checklist‐based tool for quality appraisal in health‐related qualitative evidence syntheses, and is endorsed by the Cochrane Qualitative and Implementation Methods Group (Long et al., 2020). Each of the 10 items focus on a different methodological aspect of a qualitative study. To improve the tool's sensitivity to theoretical validity, the following question was added by Long et al. (2020): “*Are the study's theoretical underpinnings (e.g., ontological and epistemological assumptions; guiding theoretical framework(s)) clear, consistent and conceptually coherent?*” The CASP checklist questions were scored independently as “yes” (1), “no” (0), “somewhat” (0.5), or “cannot tell” (0) by two authors (N.C. and G.P.), and assigned a total score (out of 10). Cohen's *κ* was calculated to determine interrater reliability, showing moderate agreement (76%) between total scores (*κ* = 0.712, *p* < .001). Four studies with differences of one point were independently scored by a third author (A.T.), who rated all in line with either GP or NC, and the majority score was assigned.

## 3 RESULTS

### 3.1 Paper selection

**FIGURE 1:**
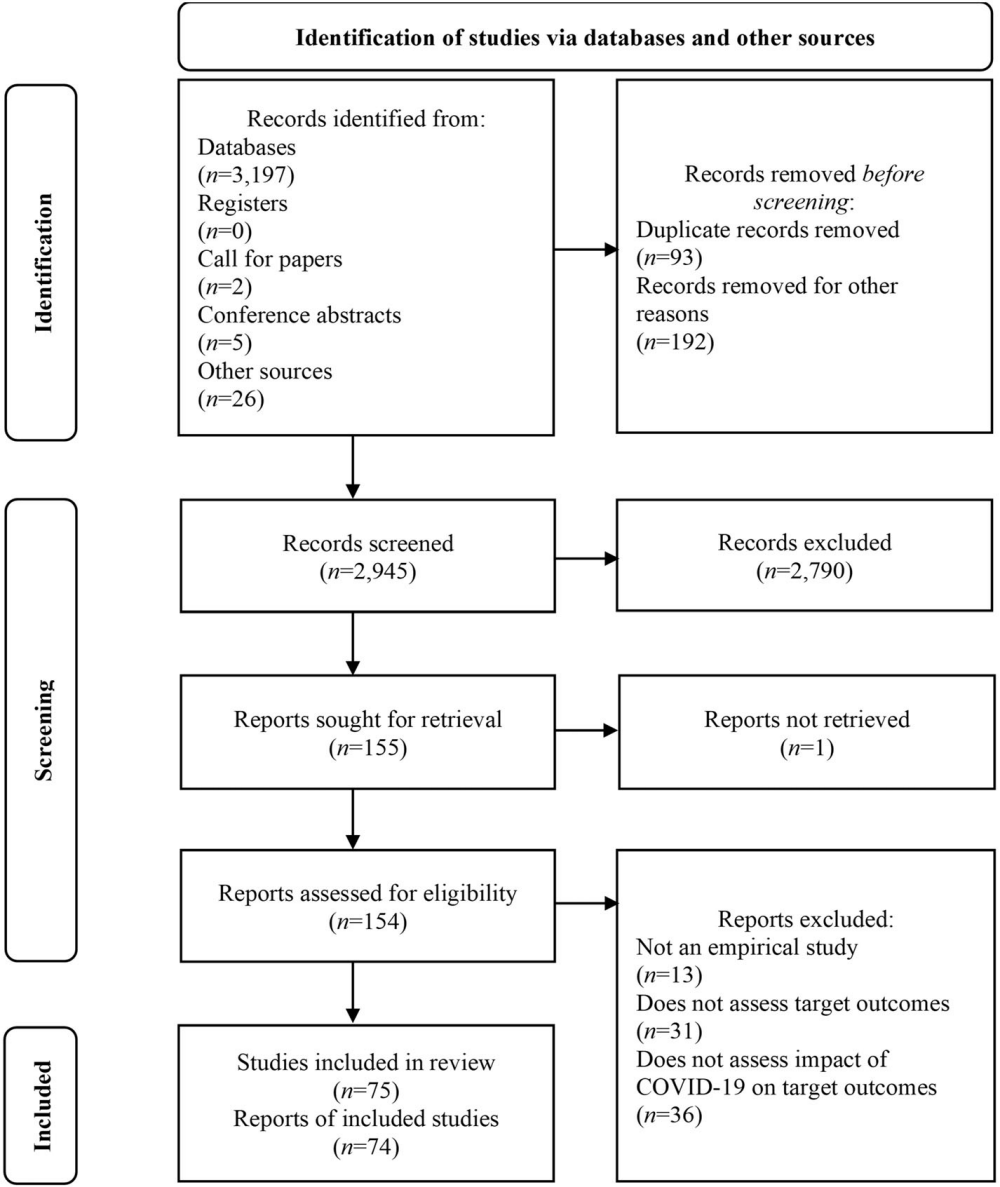
PRISMA flowchart of study selection

As of August 1, 2021, the search protocol yielded 3230 papers (see Figure 1). After removing duplicates (*n* = 93) and incomplete or nonrelevant records (*n* = 192), 2945 papers were screened based on title and abstract. Of these, 2790 were excluded and 155 were sought for retrieval, of which one report could not be retrieved. In total, 154 articles were assessed for eligibility. Thirteen papers were excluded because they did not describe an empirical study, 31 studies were excluded because they did not assess target outcomes, and 36 studies were excluded because they did not assess the influence of COVID‐19 on target outcomes.

### 3.2 Study characteristics

A final sample of 74 reports, describing 75 studies (quantitative *n* = 58, mixed‐methods *n* = 7, qualitative *n* = 10), was included in this review. Characteristics of quantitative studies (including quantitative data from mixed‐methods studies) are described in Table 1 and characteristics of qualitative studies (including qualitative data from mixed‐methods studies) are described in Table 2. Of the 65 studies that reported quantitative findings, 46 were cross‐sectional (of which 13 were retrospective), 16 were longitudinal, and 3 were cohort studies. Of the 17 studies that reported qualitative findings, six used online surveys for data collection, six used semi‐structured interviews, one used focus groups, one used a combination of semi‐structured interviews and autoethnography, one used a combination of online surveys and semi‐structured interviews, one used textual analysis of chat transcripts, and one analyzed individual posts from the social media platform Reddit. The majority of qualitative studies analyzed the data using thematic analysis (*n* = 14), two used content analysis, and one used Foucauldian discourse analysis. The studies were conducted in more than 20 countries, predominantly in Europe and North America, including the United States (*n* = 23), the United Kingdom (*n* = 12), Spain (*n* = 7), Italy (*n* = 6); Germany (*n* = 4), Portugal (*n* = 4), Australia (*n* = 3), Canada (*n* = 3), France (*n* = 2), Lebanon (*n* = 2), Turkey (*n* = 2), Austria (*n* = 1), Belgium (*n* = 1), Greece (*n* = 1), Hong Kong (*n* = 1), Ireland (*n* = 1), Lithuania (*n* = 1), Netherlands (*n* = 1), Poland (*n* = 1), and multiple countries (*n* = 2). Participants were mostly adults, with nine studies being conducted with children and/or adolescents.

### 3.3 Study quality

Of the studies evaluated using the EPHPP, 28 were rated as “weak,” 32 were rated as “moderate,” and 5 were rated as “strong.” Most studies lacked quality in study design (e.g., bias due to allocation process) and selection bias (e.g., how representative the sample is of the target population). Of the studies assessed using the CASP, 1 was rated as “weak,” 10 were rated as “moderate,” and 6 were rated as “strong.” Most studies lacked information regarding theoretical underpinnings, including ontological and epistemological approaches, as well as sufficient consideration of the researcher–participant relationship and ethical issues.

### 3.4 Theme 1: Disruptions due to the COVID‐19 pandemic

Participants with BI and eating concerns reported both social and functional restrictions resulting from the COVID‐19 pandemic. Social restrictions included social isolation and confinement (e.g., Brown et al., 2021), increased responsibility for self and others (e.g., Brown et al., 2021), and loss of, or reduced access to, usual support networks (e.g., Vuillier et al., 2021). Functional restrictions included a lack of routine and structure or disruption to previous living routine (e.g., Clark Bryan et al., 2020; McCombie et al., 2020), changes or constraints to physical activity or exercise routine (e.g., Branley‐Bell & Talbot, 2020), and restrictions to personal freedom (e.g., Zeiler et al., 2021). Alongside restrictions to social support, individuals with EDs specifically reported that the COVID‐19 pandemic had caused reduced access or interruption to professional support and treatment, including ED services, general healthcare, and mental health services (e.g., Fernández‐Aranda, Casas, et al., 2020; Fernández‐Aranda, Munguía, et al., 2020). Moreover, some participants with EDs increased their reliance on family and friends in response to a lack of access to professional support and perceived risks of replacement telehealth approaches (e.g., Hunter & Gibson, 2021).

### 3.5 Theme 2: Variability in the improvement or exacerbation of symptoms

Findings were mixed regarding the impact of COVID‐19 on BI, DE behaviors, and EDs. Studies reported inconsistent rates of symptom deterioration and improvement during COVID‐19. For example, studies found that between 33% and 70% of participants reported worse BI during COVID‐19, 10%–26% reported improved BI, and 4%–51% reported no change in BI as a result of the pandemic. In terms of EDs, 16%–87% of participants reported worsened ED symptoms or reactivation of ED symptoms during COVID‐19, 2%–51% reported improved ED symptoms, and 8%–66% reported no change in ED symptoms as compared to prepandemic levels. In addition, there was great variation in which symptoms were most affected by the COVID‐19 pandemic, with some studies showing no differences in target outcomes from pre‐ to postlockdown (e.g., Koenig et al., 2021; Martínez‐de‐Quel et al., 2021). As such, two subthemes were generated: (1) negative outcomes of the COVID‐19 pandemic and (2) positive outcomes of the COVID‐19 pandemic. Of note, multiple participants reported experiencing both positive and negative outcomes simultaneously; however, for simplicity, negative and positive outcomes are presented separately.

#### 3.5.1 Negative outcomes of the COVID‐19 pandemic

Overall, most studies reported some negative outcomes of the COVID‐19 pandemic for individuals living with EDs and the general population. Multiple studies reported deterioration in participants' BI outcomes, such as body esteem, body dissatisfaction, weight and shape concerns, and drive for thinness (e.g., Conceição et al., 2021; Keel et al., 2020; Larkin, 2021; Nutley et al., 2021; White, 2021), as well as DE behaviors, such as dietary restriction, emotional eating, binging and purging, and compulsive exercise (e.g., Giel et al., 2021; Phelan et al., 2021; Philippe et al., 2021; Zhou & Wade, 2021). Moreover, studies showed a higher prevalence of EDs during the pandemic compared to previous years (e.g., Lin et al., 2021; Spettigue et al., 2021; Taquet et al., 2021). Patients with EDs reported reduced psychological well‐being (e.g., H. Kim, Rackoff, et al., 2021; Leenaerts et al., 2021); feeling out of control (e.g., Branley‐Bell & Talbot, 2021; Richardson et al., 2020); increased suicide ideation, suicidal thoughts, and self‐harm (e.g., Monteleone et al., 2021); higher levels of loneliness (e.g., S. Kim, Wang, et al., 2021; Schlegl, Maier, et al., 2020); increased posttraumatic stress (e.g., Nisticò et al., 2021); and changes in ED‐specific and general psychopathology (e.g., Castellini et al., 2020; Simone et al., 2021; Trott et al., 2021; Vitagliano et al., 2021).

#### 3.5.2 Positive outcomes of the COVID‐19 pandemic

Positive outcomes of the pandemic relating to BI, DE, and ED experiences included making time for self‐care (e.g., McCombie et al., 2020; Termorshuizen et al., 2020); having space away from in‐person appearance comparisons and reduced weight monitoring (e.g., Graell et al., 2020; Quathamer & Joy, 2021); and connecting virtually with friends, family, and the community (e.g., Termorshuizen et al., 2020; Zeiler et al., 2021). Patients with EDs specifically reported a reduction in ED symptomatology (e.g., Schlegl, Maier, et al., 2020); increased attempts at self‐management in recovery (e.g., Schlegl, Maier, et al., 2020); greater motivation to recover (e.g., Clark Bryan et al., 2020; Termorshuizen et al., 2020); and more time for reflection on recovery (e.g., Hunter & Gibson, 2021).

### 3.6 Theme 3: Factors associated with BI and DE outcomes

As described above, social implications of the COVID‐19 pandemic and changes in participants' physical environment were associated with both symptom deterioration and improvement. As such, several associated factors were identified to explain these discrepancies. Factors that were associated with worse BI and eating outcomes during the pandemic included psychological, individual, social, and ED‐related characteristics. Psychological variables associated with worse outcomes included higher levels of worry, rumination, loneliness, anxiety, depression, stress, psychological distress, and fear of COVID‐19 (e.g., Akgül et al., 2021; Chan & Chiu, 2021; Frayn et al., 2021; Swami, Horne, et al., 2021); comorbidity of mental health concerns and childhood trauma (e.g., Castellini et al., 2020; Chan & Chiu, 2021); insecure attachment, lower self‐directedness, and poor coping strategies (e.g., Baenas et al., 2020; Haddad et al., 2020); poor emotion regulation (e.g., Félix et al., 2021; Flaudias et al., 2020; Machado et al., 2020); higher levels of uncertainty intolerance (e.g., Scharmer et al., 2020); and greater food insecurity (e.g., Christensen, Forbush, et al., 2021).

Individual variables associated with worse outcomes included identifying as a woman (e.g., Baceviciene & Jankauskiene, 2021; Buckley et al., 2021; Serin & Koç, 2020); increased time spent online or on social media (e.g., Bellapigna et al., 2021; Vall‐Roqué et al., 2021); identifying as an ethnic minority participant (e.g., Christensen, Forbush, et al., 2021; S. Kim, Wang, et al., 2021); pre‐COVID‐19 experiences of weight stigma (e.g., Puhl et al., 2020); and higher body mass index or body weight, or reporting changes in weight during COVID‐19 (e.g., Lessard & Puhl, 2021; Stoddard, 2021). Findings were inconclusive regarding the effect of age, with some studies showing younger age to be a risk factor and older age to be a protective factor (e.g., Pikoos et al., 2020; Ramalho et al., 2021), one study showing that adolescents reported higher reactivation of ED symptoms than children (Graell et al., 2020), and one study showing no influence of age on target outcomes (Monteleone, Marciello, et al., 2021). Contrary to expectations, increased videoconferencing as a result of confinement did not predict BI or binge eating postlockdown (Gullo & Walker, 2021; Pfund et al., 2020).

Social variables associated with worse outcomes included household arguments or family conflicts (e.g., Baenas et al., 2020; Castellini et al., 2020); change in living situation and access to usual support networks (e.g., Branley‐Bell & Talbot, 2020; Monteleone, Cascino, Marciello, et al., 2021); fear for the safety of loved ones (e.g., Castellini et al., 2020); longer social isolation and confinement (e.g., Coulthard et al., 2021; Haddad et al., 2021); perceived low quality of personal and therapeutic relationships (e.g., Cecchetto et al., 2021; Monteleone, Cascino, Marciello, et al., 2021); and exposure to COVID‐19‐related media and triggering messages regarding quarantine weight gain and exercise (e.g., Nutley et al., 2021; Vuillier et al., 2021). Evidence was mixed regarding participants' living situation, with some studies showing that living alone or living with fewer people was associated with less favorable outcomes (Félix et al., 2021; Pikoos et al., 2020), and other studies showing the detrimental effects of living with a higher number of adults in confinement (Haddad et al., 2020; Zeiler et al., 2021).

Finally, ED‐related variables associated with worse outcomes included a prior or current ED diagnosis (e.g., Breiner et al., 2021; Meda et al., 2021; Phillipou et al., 2020; Robertson et al., 2021) and higher levels of BI and/or eating concerns at baseline (e.g., Jordan et al., 2021; Pfund et al., 2020). Results were inconclusive regarding ED subtype. Two studies found that patients with anorexia nervosa (AN; Baenas et al., 2020; Monteleone, Marciello, et al., 2021) and other specified feeding or eating disorders (OSFED; Baenas et al., 2020) were more likely to report symptom deterioration and worse mental health outcomes during confinement. In addition, patients with AN reported the greatest dissatisfaction and accommodation difficulty with remote therapy (Fernández‐Aranda, Munguía, et al., 2020). However, another study found that patients with BN reported more severe COVID‐19‐related posttraumatic symptomatology than patients with AN and healthy controls (Castellini et al., 2020). Similarly, patients with AN reported a positive response to treatment during confinement, while no changes were found in patients with BN, and patients with OSFED reported an increase in eating symptomatology and psychopathology (Fernández‐Aranda, Munguía, et al., 2020). In terms of prevalence, one study found that increased diagnostic incidence of EDs in 2020 was primarily related to AN (Taquet et al., 2021), while another study showed increased prevalence of BN and BED during the pandemic, with no differences found in prevalence of AN from pre‐ to during COVID‐19 (H. Kim, Rackoff, et al., 2021). However, it should be noted that Taquet et al.'s study included health records of more than 5 million people to compare ED incidence risk in 2020 with previous years, while H. Kim, Rackoff, et al. (2021) longitudinally examined change in frequencies of psychological health conditions in a sample of 4970 participants from pre‐ to during COVID‐19, making comparison between studies difficult. Finally, several studies showed no effect of ED diagnosis on target outcomes (e.g., Monteleone, Cascino, Marciello, et al., 2021; Vuillier et al., 2021).

Multiple factors were also identified that were associated with more positive and less negative BI and eating outcomes during the pandemic, including personal characteristics, such as adaptive coping mechanisms (e.g., Baenas et al., 2020), self‐compassion (e.g., Swami, Todd, et al., 2021), emotion regulation (e.g., reappraisal; Giel et al., 2021), sense of coherence (e.g., Giel et al., 2021), and higher perceived control (e.g., Branley‐Bell & Talbot, 2020); social characteristics, such as emotional and social support from others (e.g., Tabler et al., 2021), virtual social contact with friends and family (e.g., Schlegl, Meule, et al., 2020), and more time spent with family and improved family relationships (e.g., Vuillier et al., 2021); and individual behaviors, such as mild physical activity (e.g., Schlegl, Meule, et al., 2020), taking part in enjoyable activities (e.g., Schlegl, Maier, et al., 2020; Schlegl, Meule, et al., 2020), maintaining daily routines (e.g., Schlegl, Maier, et al., 2020), and day planning (e.g., Schlegl, Maier, et al., 2020).

Notably, findings were inconsistent regarding the influence of physical activity on BI and eating outcomes. Some studies have suggested that *change* in an individuals' physical activity routine as a result of restrictions may be associated with worse outcomes, regardless of their current level of physical activity. For example, Martínez‐de‐Quel et al. (2021) found that the lockdown period due to COVID‐19 negatively influenced physical activity levels, sleep quality, and well‐being in participants who had a physically active lifestyle before the COVID‐19 pandemic, but not in participants who were classed as physically inactive prepandemic. However, they did not assess the relationship between changes in physical activity levels and ED risk. Two studies found that change in physical activity was associated with worsened ED symptoms (Branley‐Bell & Talbot, 2020; Vuillier et al., 2021). Specifically, for some people, reduced physical activity as a result of restrictions was associated with increased ED cognitions or compensatory disordered behaviors (e.g., food restriction), while others engaged in more excessive exercise at home to cope with the loss of their usual physical activity routine. Indeed, several studies found that physical activity was positively associated with adverse outcomes in participants with EDs and in the general population, such as dietary restraint, eating concerns, shape and weight concerns, and eating symptomatology (Haddad et al., 2020; Monteleone, Marciello, et al., 2021).

### 3.7 Theme 4: Unique challenges for marginalized and underrepresented groups

In line with the aim to highlight the impact of the COVID‐19 pandemic on BI and DE outcomes in marginalized and underrepresented populations, the final theme relates to findings from studies that have included such participant samples. Specifically, we considered the influences of sexuality, gender identity, belonging to a racialized group, and ethnicity. As the majority of studies were conducted in Europe or North America, we were not able to explore differences in the effect of COVID‐19 on target outcomes in countries that are typically less represented in psychological research (i.e., non‐WEIRD countries).

Among studies that reported race or ethnicity data (*n* = 32; 43%), a majority of participants were White, with few studies comparing outcomes between participants who identified with different ethnicities or racialized groups. S. Kim, Wang, et al. (2021) found that identifying as Black was associated with lower psychological distress, stress, and loneliness, while identifying as Asian was associated with higher psychological distress, stress, and loneliness in participants with confirmed or suspected EDs. Moreover, Christensen, Forbush, et al. (2021) found that participants who identified as Black were significantly more likely to report individual food insecurity relative to other racialized groups, but found no differences in food insecurity from before to during the beginning of the COVID‐19 pandemic. Overall, participants who identified as BIPOC reported similar BI and eating concerns as outlined in previous themes.

Only nine studies (12%) reported participants' sexual orientation and 19 studies (25%) included participants with nonbinary or transgender identities. Of note, the majority of the included studies did not specify whether they assessed sex assigned at birth, gender, or gender identity, and many used “sex” and “gender” interchangeably. In addition, most studies included predominantly cisgender and heterosexual participants, apart from Quathamer and Joy (2021), who specifically explored the impact of the COVID‐19 pandemic on BI among LGBTQ+ individuals in Canada, and Tabler et al. (2021), who investigated the impact of COVID‐19 on self‐reported ED symptoms and perceived weight gain among LGBTQ+ adults in the United States. None of the studies included in this review directly investigated the unique experiences of BI, DE, or EDs during COVID‐19 among individuals who identified as nonbinary or transgender. Of the 19 studies that reported the inclusion of nonbinary and transgender participants, only four studies conducted analyses based on gender, of which two excluded these participants due to small group size (<6%), instead focusing on differences in target outcomes within the male–female gender binary. In addition, one study grouped all participants who identified as LGBTQ+ in their analyses. Indeed, even in studies that specifically targeted LGBTQ+ samples (Quathamer & Joy, 2021; Tabler et al., 2021), the majority of participants identified as cisgender. As such, findings across all participants who identified as LGBTQ+ are presented together in this review, while we acknowledge the possible differences in experiences across various identities.

Overall, findings indicate that pandemic‐related stress was associated with ED symptoms and perceived weight gain, and this association was stronger in women and individuals who identified as LGBTQ+ compared to cisgender and heterosexual men (Tabler et al., 2021). Although findings regarding the impact of the COVID‐19 pandemic on BI and eating outcomes generally mirrored those discussed above, individuals who identified as LGBTQ+ discussed challenges of COVID‐19 through a lens of gender identity and sexuality. For example, some participants reported unique challenges directly related to restrictions imposed as a result of the COVID‐19 pandemic, such as being confined with family members who were not aware of, or did not approve of, their gender identity or sexuality (Quathamer & Joy, 2021). In addition, participants reported gender dysphoria‐related body issues due to the fear of gaining weight during quarantine (Quathamer & Joy, 2021). On the other hand, several participants reported that the restrictions associated with the pandemic gave them a break from the pressure to present their body and gender in a socially acceptable way (Quathamer & Joy, 2021). Some participants further reported that the removal of distractions and a break from engaging with a heteronormative society allowed them to be present more fully in their body and explore their gender identity in greater depth (Quathamer & Joy, 2021). Of note, several studies reported no effect of gender (e.g., H. Kim, Rackoff, et al., 2021; Puhl et al., 2020), ethnicity/racialized group belonging (e.g., Coulthard et al., 2021; Czepczor‐Bernat et al., 2021; H. Kim, Rackoff, et al., 2021), or sexual orientation (e.g., Czepczor‐Bernat et al., 2021; H. Kim, Rackoff, et al., 2021) on BI or eating outcomes during COVID‐19.

## 4 DISCUSSION

The current review is the first to investigate the influence of the COVID‐19 pandemic and related restrictions on BI, DE behaviors, and ED outcomes. The findings of this review complement and extend findings from previous reviews conducted on the impact of COVID‐19 on EDs (Miniati et al., 2021; Monteleone, Cascino, Barone, et al., 2021; Sideli et al., 2021). First, previous reviews evaluate earlier research on COVID‐19 (spanning the time period from January 2020 to January 2021). Our review extends this time period until August 2021, which includes the easing of restrictions in many countries. Second, previous reviews have included between 21 and 26 papers, most of which were quantitative, and have predominantly focused on ED outcomes. We include 75 studies (including 16 longitudinal studies and 17 studies that report qualitative findings) and adopt a mixed‐studies approach to explore both quantitative and qualitative outcomes. Moreover, the current review contributes novel findings on the impact of COVID‐19 on BI and DE behaviors in the general population. As such, we also extend existing recommendations beyond clinical practice and treatment (see Table 4). Four themes were generated across qualitative, quantitative, and mixed‐methods studies, including: (1) disruptions due to the COVID‐19 pandemic; (2) variability in the improvement or exacerbation of symptoms; (3) factors associated with BI and DE outcomes; and (4) unique challenges for marginalized and underrepresented groups.

**TABLE 4:** Recommendations for individuals, researchers, clinicians, and public health messaging

Important findings	Recommendations for individuals	Recommendations for researchers	Recommendations for clinicians	Recommendations for public health messaging
Weight stigma/stigmatizing messaging: COVID‐19‐related media and triggering messages regarding quarantine weight gain and exercise were found to negatively influence body image and eating outcomes (e.g., Nutley et al., 2021; Vuillier et al., 2021). Pre‐COVID‐19 experiences of weight stigma were associated with more negative outcomes (e.g., Puhl et al., 2020).	Reduce time spent online, particularly if the media content makes you feel worse about your body. Be critical about media reporting worse health outcomes in individuals with higher weight in recognition of widespread societal anti‐fat weight bias and an overreliance on correlational data to imply causation.	Qualitative and quantitative research is required to assess the potentially harmful effects of “antiobesity” messaging on individuals' mental health and well‐being during the pandemic and beyond. This research should take into account a variety of media promoting such messages, including formal (e.g., news outlets, articles) and informal media (e.g., social media posts, memes). This research should consequently be used to inform public health messaging guidelines to avoid stigmatizing language and promote size‐ and weight‐inclusive health messaging.	Explore client's social media use (e.g., frequency, duration, accounts followed). Work together to identify helpful and unhelpful content, the costs/benefits of (dis)engaging with this content and how to cultivate a helpful social media environment (e.g., unfollowing accounts, reporting harmful content). Due to the high proportion of individuals reporting increased exposure to weight stigmatizing social media content during COVID‐19, and the negative impact of weight stigmatizing public health campaigns on mental and physical health, clinicians should adopt a weight‐neutral approach, such as Health at Every Size.	Use nonstigmatizing and weight‐inclusive alternatives to health messaging around COVID‐19. When designing a health message, image choice (i.e., weight‐inclusive rather than weight‐stigmatizing) and wording (i.e., non‐forceful) should be considered. Health messaging should focus on specific, *realistically achievable* behaviors, rather than weight or appearance (e.g., engaging in physical activity, keeping a safe distance, handwashing) and encourage activities for mental health and well‐being as well (e.g., spending time with family and friends, taking breaks, trying new hobbies).
Disruptions/changes to treatment: Most patients reported a disruption in treatment or professional support during the pandemic (e.g., Fernández‐Aranda, Casas, et al., 2020; Fernández‐Aranda, Munguía, et al., 2020). Different patients respond differently to online treatment (e.g., Hunter & Gibson, 2021). The inability to fully access professional services was highlighted as a major concern for those participants receiving professional support prior to the pandemic/confinement period (e.g., Hunter & Gibson, 2021).	Do not hesitate to seek support and treatment, and try to find something that works for you, whether it is in person, online, or via telephone. If in‐person services are not currently available, we encourage you to be open to trying different modalities of treatment. Online treatment might not work for everyone, but some people might prefer it. Communicate with your therapist or support provider about any challenges you are facing with the available treatment options and let them know about any modifications you require to adjust the treatment to suit your individual situation.	Research is required to investigate for whom online treatment works best and how to adapt online treatment to enhance effectiveness and patient acceptability. This research should consequently be used to inform treatment and support adaptations, and feed into novel telehealth approaches to ensure treatment is both accessible and effective, including for individuals who have difficulties accessing in‐person support due to the pandemic or other reasons.	When adapting or moving treatment programs to online platforms, consider participant preferences and living conditions (e.g., privacy, access to safe spaces). Offer patients time to share their thoughts and concerns about changes in treatment routine/methods. This should be at the start of the session and not derail the treatment plan and goals. If treatment is modified for existing patients, clear and timely communication about when and how their regular treatment will be modified is required. For patients with more advanced symptoms that include secretive disordered behaviors, in‐person therapy is preferred to enhance accountability and transparency. Going forward, clinicians may consider adopting hybrid or flexible working practices, considering individual patients' needs and preferences, especially as COVID‐restrictions continue to be eased in many countries.	Messaging should be clear and timely regarding available treatment providers and alternative support options, such as telehealth or online therapy. Messaging should also be accessible for everyone (e.g., not dependent on access to the internet) and easy to follow.
Lack of access to personal/professional support: Change in living situation and/or access to usual social and professional support networks were associated with more negative and less positive outcomes during the COVID‐19 pandemic (e.g., Branley‐Bell & Talbot, 2020; Monteleone, Cascino, Marciello, et al., 2021).	Continue engaging in contact with family and friends, even if it is virtual; try to stay in contact with people who are important to you. Try to avoid people who make you feel worse about your body or food choices.	More research is required on the impact of increased videoconferencing, while simultaneously spending less time seeing people and worrying about physical appearance in person, as well as the protective and risk factors that may predict increased appearance concerns; this is particularly important as flexible or remote working is likely to continue in the future.	Assist your patients in building online and in‐person social support networks if these have been disrupted during the pandemic; for example, by building connections with local communities (e.g., schools, community centers) to facilitate patient connectedness. This may be most applicable for patients transitioning from in‐patient/day‐patient treatment.	
Physical activity/daily routines: Engaging in some physical activity may protect against negative outcomes during the COVID‐19 pandemic (e.g., Schlegl, Meule, et al., 2020). Taking part in enjoyable activities and maintaining daily routines were associated with more positive and less negative outcomes during COVID‐19 (e.g., Schlegl, Maier et al., 2020; Schlegl, Meule et al., 2020). Additionally, some individuals reported improved eating disorder symptoms due to having more time to engage in self‐care activities (e.g., McCombie et al., 2020; Termorshuizen et al., 2020).	Mild physical activity may be beneficial, but monitor your thoughts and motivations for exercise if this has previously been triggering for you; this can include dance, stretching, yoga, walking, or other types of exercise that you enjoy. Try to maintain or create new daily routines by planning your day or week in advance, and make sure to incorporate some enjoyable activities into your day. Moreover, take time for self‐care, whether it is spending time alone, reading a book, meditating, painting, listening to music, or learning something new.	More research is required on the protective benefits of physical activity during lockdown, depending on physical activity type (e.g., vigorous versus mild) and specific population (e.g., patients with eating disorders versus healthy participants). More research is required to understand individual differences in experiences of, and responses to, COVID‐19 restrictions, as well as to identify risk and protective factors or mechanisms that can be targeted in future interventions and treatment approaches. Such research should (1) employ longitudinal or cohort study designs and (2) investigate risk and protective factors at multiple levels (i.e., individual, social, societal).	Tailor physical activity advice to individual patients' needs and discuss any changes in exercise routine caused by the pandemic. Discuss coping strategies with your patients and identify what strategies are most beneficial to help them in their recovery. For example, help your patients create and maintain a daily routine if it has been disrupted by the pandemic. Encourage your patients to spend some time on self‐care activities, or activities that they enjoy and that make them feel good.	
Impact on marginalized groups: There is some evidence showing that participants who identify as LGBTQ+ and/or BIPOC are more at risk of reporting adverse mental health, body image, and eating outcomes during COVID‐19 (e.g., Christensen, Forbush, et al., 2021; S. Kim, Wang, et al., 2021; Tabler et al., 2021; Quathamer & Joy, 2021). Limited research has been conducted on the impact of the COVID‐19 pandemic on participants from historically marginalized or underrepresented groups.		More research is required on minoritized, racialized, underrepresented, or otherwise marginalized participants during COVID‐19, considering the effects of intersectionality (i.e., the connectedness and interaction between multiple identities) and inequality (e.g., biases, discrimination, stigma) on body image and eating concerns. Additionally, more research is required on groups previously underrepresented in eating disorder research (e.g., people of color, men) and individuals who may have unique body image and eating concerns (e.g., individuals who identify as LGBTQ+, adolescents). Researchers should consider targeted recruitment strategies to reach underresearched populations and distinguish participants based on more than one identity factor when presenting study results (e.g., Black men, Black women, White men, White women).	When working with patients from marginalized backgrounds, clinicians need to consider additional concerns and unique challenges that these participants may face (e.g., experience of discrimination), which may impact both symptomatology and treatment outcomes. Additionally, clinicians need to consider their own implicit biases and explicit behaviors when working with all patients.	Strive for inclusivity and diversity by engaging marginalized and underrepresented communities in designing public health messaging. Collaborate with researchers, clinicians, and community leaders to ensure messaging is accurate, timely, and relevant.

### 4.1 The influence of COVID‐19 on BI and DE outcomes

Overall, qualitative and quantitative data complemented each other and showed a negative influence of the COVID‐19 pandemic on BI and DE. Specifically, with respect to BI, studies showed increased shape and weight concerns (Schlegl, Maier, et al., 2020), drive for thinness/muscularity (Swami, Horne, et al., 2021), body and appearance dissatisfaction (Vall‐Roqué et al., 2021), and decreased self‐esteem (White, 2021). Worsened DE behaviors included binge eating (Zhou & Wade, 2021), dietary restriction (Termorshuizen et al., 2020), and compulsive exercise (Scharmer et al., 2020). Furthermore, specific and general symptomatology as well as mental health outcomes worsened in individuals living with EDs, including increases in stress (Fernández‐Aranda, Casas, et al., 2020), anxiety (Richardson et al., 2020), and depression (H. Kim, Rackoff, et al., 2021). The majority of participants also reported perceived disruptions of the pandemic to their daily routine (Nutley et al., 2021), social support networks (Vuillier et al., 2021), and access to treatment and professional support (Hunter & Gibson, 2021).

Conversely, many studies also reported positive outcomes of the COVID‐19 pandemic, including reduction in ED symptomatology, more time to reflect on recovery and engage in self‐care, greater motivation to recover, and more time to connect with family in person or online (e.g., McCombie et al., 2020; Schlegl, Maier, et al., 2020; Termorshuizen et al., 2020; Zeiler et al., 2021). From the, albeit limited, studies that reported socioeconomic status, the current data likely reflects participants with higher social privilege. Therefore, we speculate that, across studies, participants may have incurred fewer financial pressures, which afforded greater access to leisure time and facilities during quarantine, compared with the general population. As such, this may have facilitated engagement with self‐care, recovery strategies, and social support as discussed in the qualitative literature, thus contributing to the reduced ED symptomatology reported across several studies. Based on the findings of this review, Table 4 outlines multiple recommendations for individuals, researchers, clinicians, and public health messaging.

Collectively, the included studies identified several factors that may contribute to an individual's risk and/or protection from adverse outcomes during the pandemic. The most commonly reported factors associated with worse outcomes during the pandemic included psychological distress, comorbidity, poor coping and emotion regulation strategies, female gender, increased time spent online, longer periods of social isolation and confinement, and higher levels of BI and eating concerns at baseline (e.g., Baenas et al., 2020; Castellini et al., 2020; Coulthard et al., 2021; Haddad et al., 2020). Notably, body mass index was also reported as a correlate of adverse BI and DE/ED outcomes; however, this is likely due to the effect of societal stigma (i.e., through weight‐centric healthcare and public health messaging) and internalized weight stigma (Lessard & Puhl, 2021; Pearl & Schulte, 2021). Unsurprisingly, the most commonly reported factors associated with better outcomes were contradictory of the abovementioned factors, and included adaptive coping mechanisms and emotion regulation strategies, social support, taking part in enjoyable activities, and maintaining daily routines (e.g., Baenas et al., 2020; Branley‐Bell & Talbot, 2020; Giel et al., 2021; Schlegl, Maier, et al., 2020; Schlegl, Meule, et al., 2020).

Findings were inconsistent regarding the role of ED subtype as a risk factor for more adverse outcomes. There are several possible explanations for this. First, study findings are likely to be influenced by the type of measures used and which symptoms the measures target. In the current review, there was high variability in measures used to assess BI and eating outcomes, making comparison across studies challenging. Second, multiple studies clustered different EDs when comparing the impact of COVID‐19 on target outcomes. For example, combining participants with various EDs and comparing them to a healthy control group or comparing participants with AN to a combined participant group with BN, BED, and other EDs. Finally, it should be noted that most studies relied on self‐reporting of ED diagnosis or self‐report questionnaires of ED symptoms. Limited empirical evidence currently exists from treatment centers and clinical trials that include objective assessment of ED diagnosis and symptomatology.

### 4.2 The influence of COVID‐19 on marginalized and underrepresented populations

Very few studies have hitherto explored the influence of the COVID‐19 pandemic on participants from historically marginalized or underrepresented groups. A majority of investigations were conducted in WEIRD countries (i.e., Europe or North America), and few included participants' ethnicity or racialized group belonging, nor their sexual orientation. There was also limited consideration of how intersectionality of multiple marginalized identities may have influenced adverse mental health outcomes during the COVID‐19 pandemic. Of studies that provided information about participants' ethnicity or racialized group belonging, sexual orientation, and/or socioeconomic status, the majority of participants were White, cisgender, heterosexual, highly educated, and of a higher socioeconomic status.

There is some evidence showing that participants who identify as LGBTQ+ and/or BIPOC are more at risk of reporting adverse mental health, BI, and eating outcomes during COVID‐19 (Christensen, Forbush, et al., 2021; S. Kim, Wang, et al., 2021; Quathamer & Joy, 2021; Tabler et al., 2021). Contrary to expectations, several studies reported no effect of gender (e.g., H. Kim, Rackoff, et al., 2021; Puhl et al., 2020), ethnicity/racialized group belonging (e.g., Coulthard et al., 2021; Czepczor‐Bernat et al., 2021; H. Kim, Rackoff, et al., 2021), or sexuality (e.g., Czepczor‐Bernat et al., 2021; H. Kim, Rackoff, et al., 2021) on BI or DE. However, such studies included predominantly White, female, and heterosexual participants, which could have obscured unique effects. Future research should explicitly take into account participants' experiences of discrimination and racism as a possible mechanism of effects.

In addition, when conducting comparisons across participants, some studies combined multiple sexualities (e.g., heterosexual compared to lesbian, bisexual, queer, questioning, or other sexualities) and ethnicities (e.g., Hispanic compared to non‐Hispanic); thus, ignoring important differences between specific participant subgroups. In fact, in the majority of studies, gender, sexual orientation, and ethnicity were not reported, or were assessed as dichotomous variables (e.g., male, female; heterosexual, nonheterosexual; White, non‐White). Finally, in longitudinal studies, the proportion of LGBTQ+ and/or BIPOC participants tended to decrease over time, indicating higher attrition rates for these populations. Future studies should therefore consider employing novel strategies to ensure equitable recruitment and analysis of participant subgroups, while considering intersecting identities and the impact of experiencing multiple inequalities on mental health outcomes during COVID‐19 (Gibson et al., 2021). Furthermore, clinicians, researchers, and other practitioners should be aware of their own implicit and explicit biases, and consider how inequalities might affect their patients and/or participants with regards to symptomatology, treatment/research experiences, and outcomes (Crisp, 2021).

### 4.3 Strengths and limitations

Strengths of this review include the preregistration of the protocol with PROSPERO, the rigorous dual screening process for inclusion and quality assessment, and the use of validated and rigorous tools (EPHPP and CASP) specifically targeted for evaluation of quantitative and qualitative studies. The inclusion of qualitative, quantitative, and mixed‐methods studies allowed for a more comprehensive and global understanding of the impact of COVID‐19 on target outcomes, not limited to quantifiable measures. Finally, the insights generated from this review led to a practical recommendation guide for individuals living and/or working with EDs and those responsible for public health messaging (see Table 4).

The findings of the present review should also be considered in light of some limitations. Overall, given the range of divergent findings of studies, it is at present challenging to understand and synthesize these studies in more depth. Although presenting multiple advantages above traditional systematic reviews, mixed‐studies reviews may compound the methodological challenges of selecting, appraising, and synthesizing quantitative and qualitative research with the added difficulty of integrating the data in a meaningful way. Although we followed predetermined inclusion and exclusion criteria and multiple authors were engaged in the study selection and data extraction processes, we did not assess interrater reliability. As such, a limitation is that the accuracy of the study selection and data extraction procedures in the current review were not evaluated. Furthermore, no consensus currently exists regarding at which point and in which manner quantitative and qualitative components should be integrated, although existing guidelines were used to minimize this limitation. In addition, mixed‐studies reviews present theoretical and methodological challenges of bringing together differently structured studies, addressing different, yet related, questions, and studies conducted within different paradigms (Grant & Booth, 2009). For the current review, we adopted the convergent integrated approach (Hong et al., 2017) to ensure that the data from qualitative and quantitative studies were combined and evaluated using an established methodology to increase the richness and robustness of the synthesis (Grant & Booth, 2009; Stern et al., 2020).

## 5 CONCLUSION

The findings of this review show both negative and positive influences of the COVID‐19 pandemic and related restrictions on individuals' BI, DE outcomes, ED symptomatology, and overall mental health and well‐being. There is currently high variability in study designs, measures used, and findings across different studies and participant samples, precluding firm conclusions regarding the impact of COVID‐19 on target outcomes, particularly in the long term. However, the combined findings of this review highlight multiple important considerations for future research, including the need to identify risk and protective factors to enhance BI and eating outcomes, as well as overall mental health during the ongoing pandemic and beyond.

## AUTHOR CONTRIBUTIONS

**Jekaterina Schneider:** Conceptualization; formal analysis; investigation; methodology; project administration; writing – original draft; writing – review and editing. **Georgina Pegram:** Formal analysis; methodology; writing – original draft; writing – review and editing. **Benjamin Gibson:** Formal analysis; methodology; writing – review and editing. **Deborah Talamonti:** Conceptualization; formal analysis; methodology; writing – review and editing. **Aline Tinoco:** Formal analysis; writing – review and editing. **Nadia Craddock:** Formal analysis; methodology; writing – review and editing. **Emily Louise Matheson:** Methodology; writing – review and editing. **Mark Forshaw:** Project administration; supervision; writing – review and editing.

## CONFLICTS OF INTEREST

The authors declare no conflicts of interest.

## Data Availability

Data sharing is not applicable to this article as no new data were created or analyzed in this study.
